# Characterization of the Two *CART* Genes (*CART1* and *CART2*) in Chickens (*Gallus gallus*)

**DOI:** 10.1371/journal.pone.0127107

**Published:** 2015-05-18

**Authors:** Guoqing Cai, Chunheng Mo, Long Huang, Juan Li, Yajun Wang

**Affiliations:** Key Laboratory of Bio-resources and Eco-environment of Ministry of Education, College of Life Sciences, Sichuan University, Chengdu, P.R. China; Huazhong Agricultural University, CHINA

## Abstract

Cocaine- and amphetamine-regulated transcript (CART) peptide is implicated in the control of avian energy balance, however, the structure and expression of *CART* gene(s) remains largely unknown in birds. Here, we cloned and characterized two *CART* genes (named *cCART1* and *cCART2*) in chickens. The cloned c*CART1* is predicted to generate two bioactive peptides, cCART1(42-89) and cCART1(49-89), which share high amino acid sequence identity (94-98%) with their mammalian counterparts, while the novel *cCART2* may produce a bioactive peptide cCART2(51-91) with 59% identity to cCART1. Interestingly, quantitative RT-PCR revealed that c*CART1* is predominantly expressed in the anterior pituitary and less abundantly in the hypothalamus. In accordance with this finding, cCART1 peptide was easily detected in the anterior pituitary by Western blot, and its secretion from chick pituitaries incubated *in vitro* was enhanced by ionomycin and forskolin treatment, indicating that cCART1 is a novel peptide hormone produced by the anterior pituitary. Moreover, *cCART1* mRNA expression in both the pituitary and hypothalamus is down-regulated by 48-h fasting, suggesting its expression is affected by energy status. Unlike *cCART1*, *cCART2* is only weakly expressed in most tissues examined by RT-PCR, implying a less significant role of *cCART2* in chickens. As in chickens, 2 or more *CART* genes, likely generated by gene and genome duplication event(s), were also identified in other non-mammalian vertebrate species including coelacanth. Collectively, the identification and characterization of *CART* genes in birds helps to uncover the roles of CART peptide(s) in vertebrates and provides clues to the evolutionary history of vertebrate *CART* genes.

## Introduction

Cocaine- and amphetamine-regulated transcript (CART) encoded by a *CART* gene was originally identified in rat striatum by PCR differential display after cocaine and amphetamine administration in 1995 [1]. Two types of *CART* mRNA transcripts generated by alternative mRNA splicing have been identified in rats, and they can encode two proCARTs of different lengths: proCART1-102 (long form) and proCART1-89 (short form) [1]. However, in humans, only the short proCART1–89 has been found [2,3]. In rats, proCART has been reported to be capable of producing two bioactive CART peptides: CART(42–89) (48 amino acids) and CART(49–89) (41 amino acids) after proteolytic processing [4].

Originally, CART has been viewed as an anorectic peptide in the regulation of feeding and energy balance in mammals [5–7]. Intracerebroventricular (icv) administration of CART peptide inhibits normal and starvation-induced food intake, and completely abolishes feeding response induced by neuropeptide Y (NPY) in rats [5]. Consistent with its anorexic role in food intake, CART is reported to be abundantly expressed in many hypothalamic nuclei associated with feeding behavior, such as the arcuate nucleus (ARC), paraventricular nucleus (PVN), and lateral hypothalamus (LHA) [8]. Besides its abundant expression in the hypothalamus, CART is also widely expressed in the central nervous system (CNS)[8–10], peripheral nervous system (PNS), and other peripheral tissues of mammals, including the pituitary, adrenal gland, pancreas, ovary, and adipose tissue [4,11–19], and thus CART peptide has recently been suggested to control many other physiological processes, such as stress response, drug reward and reinforcement, anxiety, depression, reproduction, pancreatic islet β-cell development and function, and bone remodeling [20–31]. In addition, CART peptide is also present in the blood of rats and monkeys, and its concentration displays diurnal variations which is partially influenced by circulating corticosteroids [17,32,33]. This finding, together the abundant expression of CART in the pituitary [4,16], implies that CART may function as a circulating hormone as well, possibly secreted from the anterior pituitary. This notion, however, has not received much attention [17,33].

As in mammals, icv injection of CART peptide inhibits food intake in goldfish [34,35], and food deprivation decreases *CART* mRNA levels in the hypothalamus or whole brain of several teleost fish [35–37], indicating that the roles of CART peptide in feeding control and energy homeostasis is ancient and well conserved across vertebrates. Interestingly, unlike a single *CART* gene in mammals, multiple *CART* genes have been identified in several teleost fish [36], including goldfish [35], zebrafish [37], and medaka [38]. For instance, in medaka, 6 *CART* genes have been identified and found to be mainly expressed in brain tissues and several extra-brain tissues; in zebrafish, four *CART* genes have been identified, and their expressions in adult brain appear to be down-regulated by 3-day food starvation [37]. These findings raise a fundamental question, whether multiple *CART* genes exist and play similar roles in energy balance in other non-mammalian vertebrates, such as birds.

As in mammals and goldfish, icv injection of mouse CART peptide is reported to inhibit food intake in chickens [39,40]. However, the information regarding the structure, tissue expression, and the physiological roles of avian *CART* genes remains largely unknown. Therefore, using the chicken as an experimental model, our present study aims to: 1) clone the *CART* gene(s) and examine their spatio-temporal expression patterns; 2) investigate *CART* gene expression in response to food deprivation. Our results clearly show that two *CART* genes, named *cCART1* and *cCART2*, co-exist in chickens. Interestingly, cCART1 is detected to be predominantly expressed in the anterior pituitary, and less abundantly in the hypothalamus, and its expression at both sites could be down-regulated by fasting. Our findings not only support an association between hypothalamic and pituitary cCART1 expression and nutritional status in birds, but also illustrate a clear concept that CART1 is a novel pituitary hormone in chickens. Despite the possibly less significant role of *CART2* in chicken tissues implied by its relatively weaker expression to its paralog, the existence of this novel *CART2* gene in chickens, together with the identification of *CART2* and other novel *CART* genes in lower vertebrates, such as coelacanths (6 *CART* genes), tilapia (7 *CART* genes), and zebrafish (6 *CART* genes), provide critical clues to the evolutionary history of *CART* gene during vertebrate evolution.

## Materials and Methods

### Ethics statement

All of the animal experiments were conducted in accordance with the Guidelines for Experimental Animals issued by the Ministry of Science and Technology of People’s Republic of China. The experimental protocol used in this study was approved by the Animal Ethics Committee of College of Life Sciences, Sichuan University (Chengdu, China).

### Chemicals, enzymes, primers, and antibodies

All the chemicals were obtained from Sigma-Aldrich (St. Louis, MO) and restriction enzymes were obtained from Takara (Takara, Dalian, China) unless stated otherwise. Ionomycin was purchased from Cayman chemical company (Ann Arbor, MI), Phorbol-12-myristate-13-acetate (PMA) and forskolin were from Calibiochem (Merck KGaA, Darmstadt, Germany). Rabbit anti-CART polyclonal antibody [CART(H-47), sc366086] was purchased from Santa Cruz Biotechnology Inc (Santa Cruz Biotechnology Inc, Dallas, TX), and monoclonal antibody for β-actin was from Cell Signaling Technology Inc. (CST, Beverly, MA). All primers used in this study were synthesized by Beijing Genomics Institute (Shanghai, China) and listed in S1 Table.

### Total RNA extraction

Adult Lohmann layer chickens, chicks, and chicken embryos at embryonic day (E) 8, E12, E16, and E20 were purchased from local commercial company or market. Chickens were euthanized by decapitation, and different tissues including whole brain, heart, duodenum, kidneys, liver, lung, muscle, ovary, testes, pituitary, spleen, pancreas, and various brain areas (including telencephalon, midbrain, hindbrain, cerebellum, and hypothalamus) were collected, frozen in liquid nitrogen, and stored at -80°C until use. Total RNA was extracted from chicken tissues by using RNAzol (Molecular Research Center, Cincinnati, OH) according to the manufacturer’s instructions and dissolved in diethyl pyrocarbonate (DEPC)-treated H_2_O.

### Reverse transcription and polymerase chain reaction (RT-PCR)

Oligodeoxythymide (0.5 μg) and total RNA (2 μg) isolated from different tissues were mixed in a total volume of 5 μL, incubated at 70°C for 10 min, and cooled at 4°C for 2 min. Then, the first strand buffer, 0.5 mM each deoxynucleotide triphosphate, and 100 U Moloney murine leukemia virus (MMLV) reverse transcriptase (Takara) were added into the reaction mix in a total volume of 10 μL. Reverse transcription (RT) was performed at 42°C for 90 min. All RT negative controls were performed under the same condition, but without the addition of reverse transcriptase.

According to our previously established method [41], RT-PCR assay was performed to examine mRNA expression of *cCART1* and *cCART2* genes in chicken tissues. PCR was performed under the following conditions: 2 min at 95°C denaturation, followed by 23 cycles (for *β-actin*: 30 sec at 95°C, 30 sec at 56°C, and 45 sec at 72°C) and 35 cycles (for chicken *CART1*: 30 sec at 95°C, 1 min at 62°C, 60 sec at 72°C; for chicken *CART2*: 30 sec at 95°C, 45 sec at 60°C, 60 sec at 72°C) of reaction, ending with a 5-min extension at 72°C. The PCR products were visualized on a UV-transilluminator (Bio-Rad Laboratories, Inc. Herculas, CA) after electrophoresis on 2% agarose gel containing ethidium bromide.

### Cloning of the full-length cDNAs of chicken *CART1* and *CART2* genes

According to the predicted cDNA sequence of *CART* gene (called *cCART1* here) deposited in GenBank (XM_003643097), gene-specific primers were designed to amplify the full-length cDNA containing an open reading frame (ORF) of *cCART1* gene from adult chicken brain by PCR. The amplified PCR products were cloned into pTA2 vector (TOYOBO, Osaka, Japan) and sequenced.

In addition to *cCART1* gene, a novel *CART*-like gene, designated c*CART2* herein, was also predicted in the chicken genome (XM_004950770). Since the predicted 5’cDNA end of this *CART2* gene is erroneous, therefore, using brain cDNA as the template, rapid amplification of cDNA ends (RACE) assay was performed to amplify the 5’-cDNA ends using SMART-RACE cDNA amplification Kit according to the manufacturer’s instructions (Clontech, Palo Alto, CA). The amplified PCR products were cloned and sequenced. Then, according to the *cCART2* 5’ cDNA ends, new gene-specific primers were designed to amplify the full-length cDNA sequence containing a complete ORF from adult chicken brain. The amplified PCR products were cloned into pTA2 vector (TOYOBO) and sequenced.

### Data mining, sequence alignment, and phylogenetic analysis

To determine whether multiple *CART* genes exist in other vertebrate species as in chickens, using chicken (c-)*CART1* and c*CART2* cDNA sequences as references, we performed a blast search in the 20 publicly available genomes (http://www.ensembl.org) and identified multiple copies of *CART*-like genes (2 to 7 copies) in all the non-mammalian species examined. The amino acid sequences of these *CART* genes were aligned using the ClustalW program (BioEdit, Carlsbad, CA). The putative signal peptide was predicted by using an online software [42]. Phylogenetic analysis was computed using the program MEGA5, in which the phylogenetic tree was constructed using the maximum likelihood method with 1,000 bootstrap replicates.

### Quantitative real-time PCR (qRT-PCR)

To examine the mRNA levels of c*CART1* gene in chicken tissues, quantitative real-time RT-PCR was performed. In brief, before quantitative real-time PCR assay, plasmid containing full-length cDNA of *cCART1* gene or PCR product of *β-actin* was quantified by a Spectrophotometer (Eppendorf, Hamburg, Germany), and the copy numbers of DNA molecules were calculated. Then, the serially diluted plasmids or PCR products with known copy numbers were used to set the standard curves in each quantitative real-time PCR assay. The real-time PCR was conducted on the CFX96 Real-time PCR Detection System (Bio-Rad) in a volume of 20 μL containing 0.5 μL RT product, 1 x PCR buffer, 0.2 mM each dNTP, 2.5 mM MgCl_2_, 0.2 mM each primer, 0.5 U Taq DNA polymerase (Invitrogen), and 1 μL EvaGreen (Biotium Inc., Hayward, CA). The PCR profile consisted of 40 cycles of 94°C for 20 sec, 60°C for 15 sec, 72°C for 30 sec. To assess the specificity of PCR amplification, melting curve analysis and agarose gel electrophoresis were performed at the end of the PCR reaction to confirm that a specific PCR band was produced. In addition, the identity of PCR products for all genes was confirmed by sequencing. Finally, according to the standard curve included in each quantitative real-time PCR assay, the copy numbers of target gene transcripts in RT samples were calculated.

### Effects of fasting on *cCART1* mRNA expression in the hypothalamus and pituitary

In this experiment, male chicks purchased from local company were maintained at 22°C on a 14-h light, 10-h dark (14L:10D) photoperiod and fed a commercial mixed diet with free access to water. To determine whether fasting modifies the *cCART1* expression in the hypothalamus and pituitary, twenty 2-week-old healthy male chickens of similar body weight were randomly divided into two groups: experimental group (10 individuals) and control group (10 individuals). In the experimental group, chickens had free access to water but deprived of food for 48 h. In the control group, all chickens had free access to food and water. At the end of the experiments, the pituitaries and hypothalami were collected from both groups of chickens for RNA extraction and their *cCART1* mRNA levels examined by qRT-PCR.

### Detection of *cCART1* expression and secretion in chicken anterior pituitaries by Western blot analysis

To examine the cCART1 expression and secretion in chicken anterior pituitaries, Western blot was used in this experiment, as described in our recent study [43]. In brief, the intact anterior pituitaries collected from 1-week-old chickens (or adult chickens) were washed with PBS to remove the blood cells and placed on a 48-well plate (NUNC) supplemented with 400 μl serum-free Medium 199 (Invitrogen). After 1 h incubation at 37°C, the medium was replaced by serum-free medium, and the anterior pituitaries were incubated for additional 4 h with/without drug treatment (500 nM Ionomycin, 100 nM PMA, 5 μM forskolin). Then, cCART1 peptide secreted to the incubation medium by the pituitaries was examined by Western blot using anti-CART antibody (1: 300) (note: the validation of the antibody specificity in recognizing cCART1 peptide, but not cCART2 peptide, will be described in our other forthcoming article). Parallel blotting of β-actin and cCART1 peptide in pituitary tissue lysates was also conducted.

### Data analysis

The mRNA level of *cCART1* gene was first calculated as the ratio to that of *β-actin* and then expressed as the fold difference compared to that of brain or hypothalamus, or expressed as the percentage of control group. The data were analyzed by the Student’s *t* test (for 2 groups), or by one-way ANOVA followed by the Newman-Keuls test (for comparing all pairs of groups) with the use of GraphPad Prism 5 (GraphPad Software). To validate our results, all experiments were repeated at least twice.

## Results

### Cloning the full-length cDNAs of chicken *CART1* and *CART2* genes

Based on the two predicted *CART* gene sequences deposited in GenBank (XM_003643097; XM_004950770), using RT-PCR or RACE PCR, we amplified and cloned the full-length cDNAs of the two *CART* genes from adult chicken brains. According to their evolutionary origin discussed in the text, the two *CART* genes were designated as *CART1* and *CART2* respectively in this study.

**Fig 1:**
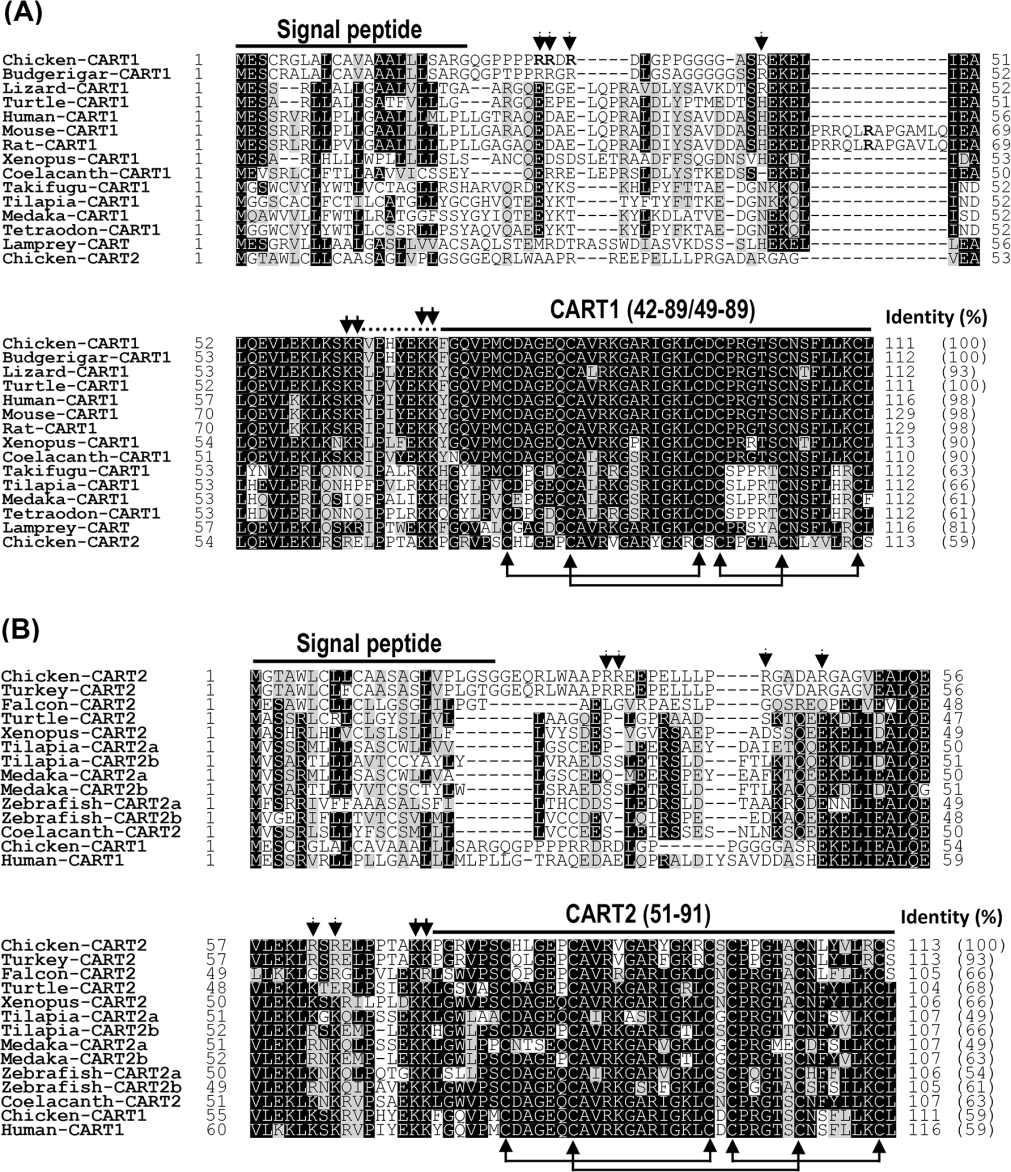
Amino acid sequence alignment of chicken CRAT1/2 with CART1/2 from other species. **(A**) Amino acid sequence alignment of chicken CART1 precursor (accession no.: KC249966) with that of budgerigars (XM_005142985), anole lizards (XM_003216266), painted turtles (XM_005288217), humans (NM_004291), mice (NM_001081493), rats (NM_017110), *Xenopus tropicalis* (XM_002934818), coelacanths (XM_006009696), takifugu, Nile tilapia, medaka (AB568295), tetraodon, or with that of lamprey CART precursor or chicken CART2 precursor (KC249967). **(B)** Amino acid sequence alignment of chicken CART2 precursor (KC249967) with that of turkeys, peregrine falcons (XM_005244209), painted turtles (XM_005278923), *Xenopus tropicalis* (XM_002935870), tilapia (CART2a: XM_003452544; CART2b: XM_003442906), medaka (CART2a: AB568293; CART2b: AB568297), zebrafish (CART2a; CART2b: GU057833), and coelacanth (XM_006002318) or with that of chicken CART1 precursor and human CART (CART1) precursor. The conserved dibasic residues (KR/KK) for proteolytic processing are marked by two arrows, while the other monobasic/dibasic residues present in chicken proCART1/proCART2 are indicated by dotted arrows. Lines linking cysteines indicate the three conserved disulfide bonds. CART sequences from species other than the ones cloned in chickens were either predicted according to their genomic sequences, or retrieved from GenBank.

The cloned chicken *CART1* (*cCART1*) cDNA consists of 3 exons (accession No.: KC249966). It encodes a precursor protein of 111 amino acids with a putative signal peptide of 22 amino acids located at its N-terminus (Fig 1). Like rat or human proCART, chicken proCART1(1–89) also contains two pairs of dibasic residues (K^40^R^41^, K^47^K^48^) critical for proteolytic processing, and thus is predicted to be capable of generating two bioactive cCART1 peptides: cCART1(42–89) and cCART1(49–89), which show remarkably high degree of identity (96–98%) with those of humans, mice, and rats. Moreover, the 6 cysteine residues critical for the formation of three disulfide bonds (Cys^55^-Cys^73^; Cys^61^-Cys^81^; Cys^75^-Cys^88^) and full biological activity of CART peptide are conserved between chicken and mammals [44–46]. In addition, cCART1(49–89) shares high amino acid sequence identity with CART1 peptide from budgerigars (100%), anole lizards (93%), painted turtles (98%), *Xenopus tropicalis* (90%), and coelacanths (90%), but a comparatively lower identity with that of takifugu (64%), Nile tilapia (66%), medaka (61%), and tetraodon (61%) (Fig 1 and Table 1).

**Table 1:** Summary of the *CART* genes identified in representative vertebrate species^a^.

Species	Gene name	Chromosome/scaffold	^d^Accession no.	Size (amino acids)
*Homo sapiens* (Human)	*CART1 (CART)*	Chr. 5	NM_004291	116
*Mus musculus* (Mouse)	*CART1 (CART)*	Chr. 13	NM_001081493	116
*Rattus norvegicus* (Rat)	*CART1 (CART)*	Chr. 2	NM_017110.1	129
*Gallus gallus* (Chicken)	*CART1*	Chr. Z	KC249966.1	111
	*CART2*	Chr. Un	KC249967.1	113
*Melopsittacus undulatus* (Budgerigar)	*CART1*	Scaffold JH556456	XM_005142985.1	112
	*CART2*	Scaffold JH556405	NA	106
*Meleagris_gallopavo* (Turkey)	*CART2*	Scaffold GL427559.1	NA	113
*Taeniopygia guttata* (Zebra finch)	*CART1*	Chr. Z	XM_002194952	Partial sequence
	*CART2*	Chr. Un	XM_002199287	Partial sequence
*Anolis carolinensis* (Anole lizard)	*CART1*	Chr. 2	XM_003216266.2	112
*Python bivittatus* (Burmese python)	*CART1*	unknown	XM_007422572.1	111
	*CART6*	unknown	XM_007429116.1	107
*Chrysemys picta bellii* (Painted turtle)	*CART1*	Scaffold JH584533	XM_005288217.2	111
	*CART2*	Scaffold JH584400	XM_005278923.1	104
	*CART3*	Scaffold JH584716.1	XM_005300628.2	109
	*CART6*	Scaffold JH584431	NA	Partial sequence
*Chelonia mydas* (Green sea turtle)	*CART1*	Unknown	XM_007059232.1	111
	*CART2*	Unknown	XM_007072062	104
	*CART6*	Scaffold 598	EMP28211	105
*Xenopus tropicalis* (Western clawed frog)	*CART1*	Scaffold GL172749	XM_002934818.1	113
	*CART2*	Scaffold GL172811	XM_002935870.2	119
	*CART3*	Scaffold GL172658	NM_001079103.1	114
	*CART4*	Scaffold GL172658	XM_002932246.2	108
*Latimeria chalumnae* (Coelacanth) ^b^	*CART1*	Scaffold JH128673.1	XM_006009696.1	110
	*CART2*	Scaffold JH127349.1	XM_006002318.1	107
	*CART3*	Scaffold JH126611.1	XM_005989732.1	112
	*CART4*	Scaffold JH126611.1	NA	108
	*CART5*	Scaffold JH128384.1	NA	116
	*CART6*	Scaffold JH128384.1	NA	Partial sequence
*Oreochromis niloticus* (Nile tilapia) ^c^	*CART1*	Scaffold GL831329.1	NA	112
	*CART2a*	Scaffold GL831251.1	XM_003452544.2	107
	*CART2b*	Scaffold GL831153.1	XM_003442906.2	112
	*CART3a*	Scaffold GL831358	XM_003456893.2	119
	*CART3b*	Scaffold GL831206	XM_003449187.2	102
	*CART4*	Scaffold GL831358	XM_005461629.1	107
	*CART5*	Scaffold GL831306	XM_003455220.2	110
*Oryzias latipes* (Japanese medaka)	*CART1*	Chr. 9	AB568295	136
	*CART2a*	Chr. 4	AB568293	107
	*CART2b*	Chr. 22	AB568297	107
	*CART3a*	Chr. 3	AB568292	118
	*CART3b*	Chr. 6	AB568294	106
	*CART5*	Chr. 11	AB568296	107
*Takifugu rubripes* (Fugu rubripes)	*CART1*	Scaffold 101	NA	112
	*CART2b*	Scaffold 10	XM_003962537.1	107
	*CART3a*	Scaffold 1	XM_003969743.1	122
	*CART3b*	Scaffold 105	XM_003967297.1	104
	*CART4*	Scaffold 1	NA	109
	*CART5*	Scaffold 274	XM_003969141.1	107
*Danio rerio* (Zebrafish) ^e^	*CART2a*	Chr. 2	NA	106
	*CART2b (CART1)*	Chr. 22	GU057833.2	105
	*CART3a (CART2b)*	Chr. 7	GU057835.2	117
	*CART3b (CART2a)*	Chr. 25	NM_001017570	120
	*CART4*	Chr. 7	NA	105
	*CART5 (CART3)*	Chr. 19	NM_001082932	105
*Tetraodon nigroviridis* (Pufferfish)	*CART1*	Chr. Un	NA	112
	*CART2*	Chr. 10	NA	108
	*CART3a*	Chr. 5	NA	Partial sequence
	*CART3b*	Chr. 13	NA	104
	*CART4*	Chr. 5	CAAE01014596.1	104
	*CART5*	Chr. Un	NA	108
*Xiphophorus maculates* (Southern Platyfish) ^c^	*CART1*	Scaffold JH556756.1	NA	109
	*CART2a*	Scaffold JH557322.1	XM_005813924.1	107
	*CART2b*	Scaffold JH556686.1	XM_005798354.1	108
	*CART3a*	Scaffold JH556875.1	XM_005808096.1	123
	*CART3b*	Scaffold JH556666.1	XM_005795806	102
	*CART4*	Scaffold JH556875.1	XM_005808120.1	107
	*CART5*	Scaffold JH557321.1	XM_005813911.1	107
*Petromyzon marinus* (Lamprey)	*CART*	Scaffold GL476395	NA	116

a. All *CART* genes were identified in the genomes of these species and the putative amino acid sequences of some *CART* genes were predicted based on their genomic sequences.

b. 6 *CART* genes, named *CART1-6*, were identified in coelacanth genome and thus, the *CART* genes identified in other representative vertebrate species were named according to their orthology to the corresponding coelacanth *CART* genes.

c. 7 *CART* genes were identified in tilapia and platyfish genomes, while only 6, or less than 6, *CART* genes were identified in the genomes of other teleost fish.

d. “NA” indicates that no predicted sequences of these *CART* genes were deposited in GenBank at the time of *in silico* data mining.

e. Two novel *CART* genes (*CART2a* and *CART4*) were identified in the present study.

The cloned chicken *CART2* (*cCART2*) cDNA consists of 3 exons (387 bp, accession no.: KC249967). It encodes a 113-amino acid precursor with a putative signal peptide of 22 amino acids at its N terminus. Like cCART1, cCART2 precursor also contains a dibasic residue (K^49^K^50^) critical for proteolytic processing, and thus it is also predicted to be capable of generating the bioactive cCART2(51–91) peptide of 41 amino acids after cleavage. Interestingly, unlike cCART1(49–89), cCART2(51–91) is much less conserved among vertebrate species examined (49–93% identity) (Fig 1). Meanwhile, it shares only 59% identity with chicken CART1(49–89) and human CART(49–89). However, the 6 cysteine residues critical for the formation of three intra-molecular disulfide bonds (Cys^57^-Cys^75^; Cys^63^-Cys^83^; Cys^77^-Cys^90^) are fully conserved among the species.

### Identification of *CART1*, *CART2*, and other *CART*-like genes in other non-mammalian vertebrates

To examine whether both *CART1* and *CART2* genes exist in other vertebrate species, using c*CART1* and c*CART2* cDNAs as references, we searched 20 representative vertebrate genome publicly available on the genome database (Table 1) including humans, mice, turtles, *Xenopus tropicalis*, coelacanths, and several teleost fishes (Nile tilapia, zebrafish, fugu, pufferfish, medaka, and platyfish). Interestingly, *CART1* and *CART2* genes were identified in non-mammalian vertebrates including teleosts. In contrast, *CART2* gene was not identified in the mammalian lineage.

**Fig 2:**
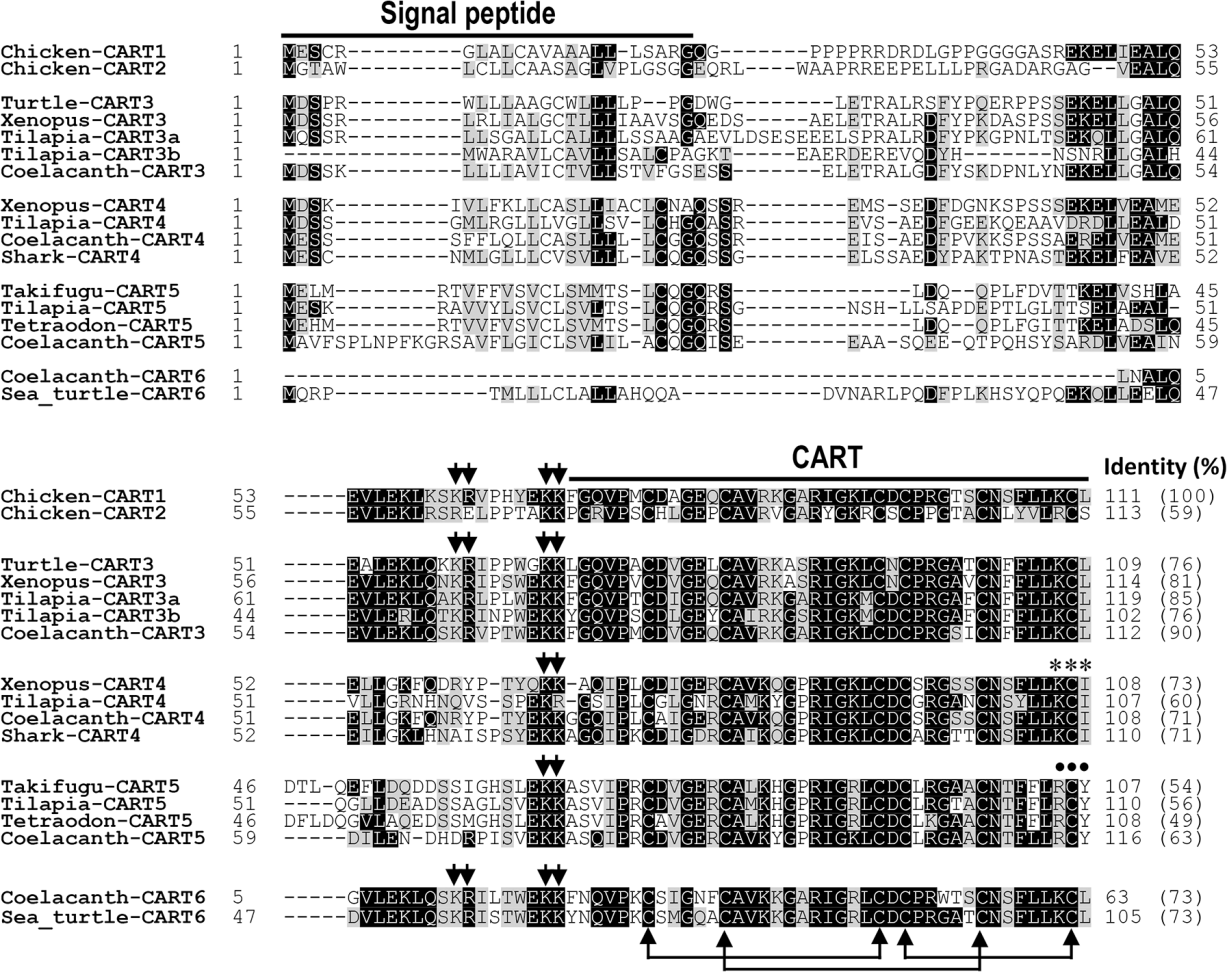
Amino acid sequence alignment of chicken CART1/2 with CART3-6 from other species. Amino acid sequence alignment of chicken CART1 (KC249966) and CART2 (KC249967) precursors with that of CART3 precursor from painted turtles (XM_005300628), *Xenopus tropicalis* (NM_001079103), tilapia (CART3a: XM_003456893; CART3b: XM_003449187) and coelacanth (XM_005989732), or CART4 precursor from *Xenopus tropicalis* (XM_002932246), tilapia (XM_005461629), coelacanth, and elephant shark (XM_007908392), or CART5 precursor from takifugu (XM_003969141), tilapia (XM_003455220), tetraodon, and coelacanth, or CART6 precursor from green sea turtles (EMP28211) and coelacanth. The conserved dibasic residues (KR/KK) for proteolytic processing are marked by two arrows presented in proCARTs. Lines linking cysteines indicate the three conserved disulfide bonds. CART sequences from species other than the ones cloned in chickens were either predicted according to their genomic sequences, or retrieved from GenBank. Asterisks and dots indicate the signature motifs (KCI or RCY) possessed by vertebrate CART4 and CART5 respectively.

**Fig 3:**
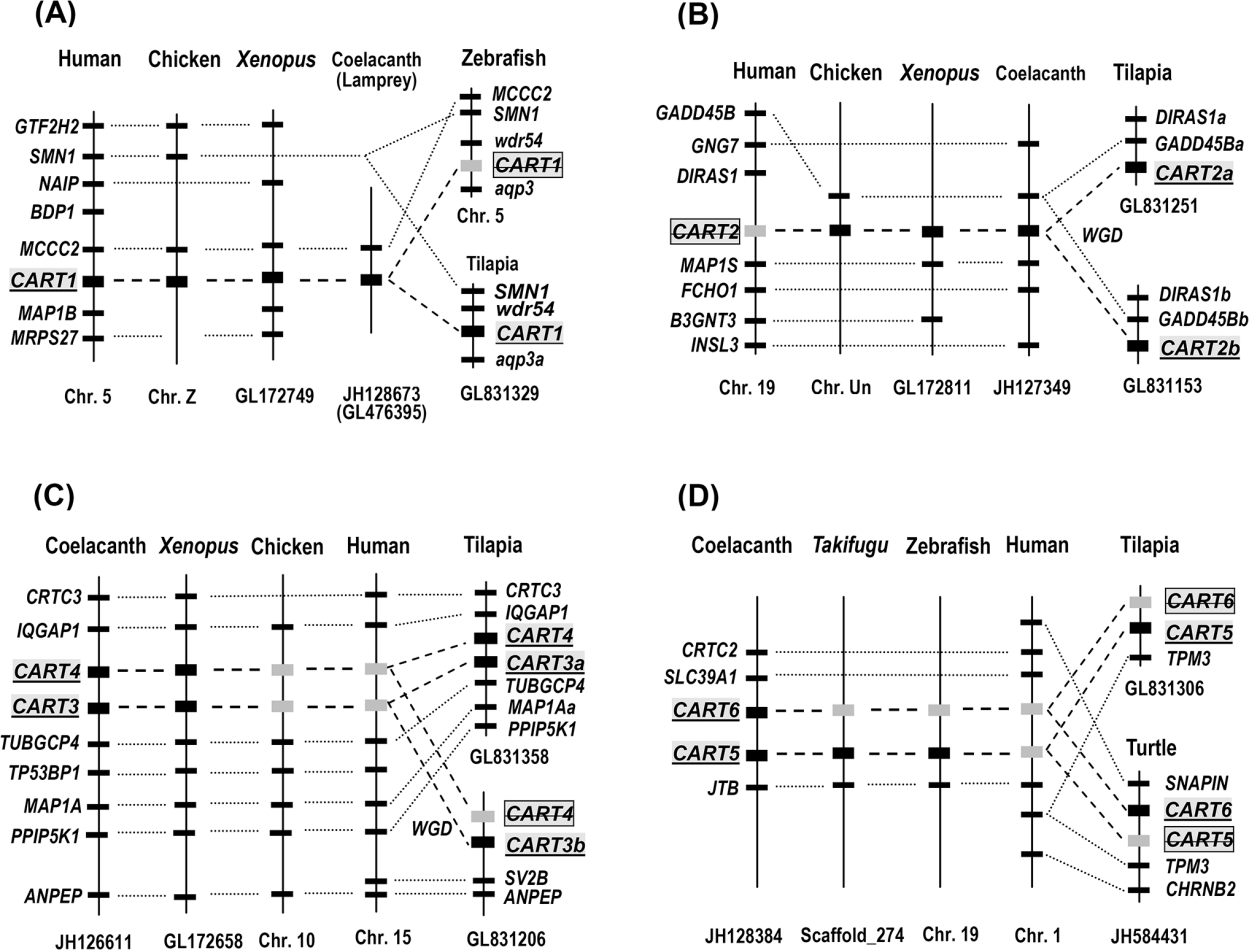
Synteny analyses of *CART* genes among species. (**A)** *CART1* gene is located in a syntenic region conserved between humans, chickens, *Xenopus tropicalis*, coelacanths, lamprey, and Nile tilapia. *CART1* was not identified in zebrafish genome. (**B**) *CART2* gene is located in a syntenic region conserved between chickens, *Xenopus tropicalis*, coelacanth, and Nile tilapia. *CART2* gene is likely lost in mammalian genomes including humans. (**C**) *CART3/CART4* genes are located in a syntenic region conserved between *Xenopus tropicalis*, coelacanth, and Nile tilapia. The two *CART* genes are likely lost in chickens and humans despite the identification of their neighboring genes. (**D**) *CART5*(/*CART6*) genes are located in a syntenic region conserved between coelacanth, takifugu, zebrafish, Nile tilapia, and painted turtles, however the *CART5/6* genes are likely lost in humans though their neighboring genes are identified. Note: the two copies of *CART2* (*CART2a* and *CART2b*) and *CART3* (*CART3a* and *CART3b*) identified in tilapia are likely to be paralogs generated by the teleost-specific whole-genome duplication (WGD) event. Dashed lines denote the genes of interest (*CART1-6*), while dotted lines indicate the syntenic genes identified in these species. Genes were labeled based on their gene symbols in the human genome. Grey bars appeared in **A**-**D** indicate the *CART* genes likely lost in these species. Chr, chromosome; Chr. Un, unknown chromosome.

In addition to *CART1* and *CART2* genes, several other *CART*-like genes were also identified in lower vertebrate species (Figs 2 and 3, S1 and S2 Figs, and Table 1). For instance, 6 *CART*-like genes were found in the genome of coelacanth (*Latimeria chalumnae*). Since all putative bioactive CART peptides encoded by the 6 *CART*-like genes share high amino acid sequence identity (49–90%) with human CART(49–89) (Figs 1 and 2), therefore, the 6 coelacanth *CART* genes were designated as *CART1*, *CART2*, *CART3*, *CART4*, *CART5*, and *CART6* herein. Among them, only *CART1* is orthologous to chicken *CART1* and human *CART*, while the other 5 novel *CART* genes are highly likely lost in humans during evolution, as suggested by synteny analysis (Fig 3). Similarly, 7 *CART* genes orthologous to coelacanth *CART* genes, named *CART1*, *CART2a*, *CART2b*, *CART3a*, *CART3b*, *CART4*, and *CART5* in this study, were identified in Nile tilapia (Fig 3) (Table 1). Among them, two copies of tilapia *CART2* (*CART2a* and *CART2b*) and *CART3* (*CART3a* and *CART3b*) were likely generated by the genome duplication event occurred in teleost lineage [47], as revealed by synteny analysis, while tilapia *CART6* gene may have been lost during evolution (Fig 3). As in tilapia, 6 or 7 copies of *CART* genes were identified in other teleost fishes, including zebrafish, takifugu, pufferfish, and platyfish (Table 1). Interestingly, 4 *CART* genes could still be identified in tetrapod species, including *Xenopus tropicalis* (*CART1*, *CART2*, *CART3*, *CART4*) and painted turtles (*CART1*, *CART2*, *CART3* and *CART6*), and one *CART* gene was identified in the genome of lamprey, an ancient vertebrate species (Table 1).

In this study, we also noted that all putative bioactive CART peptides (40/41 amino acids or 48 amino acids) encoded by 2–7 *CART* genes show 49–90% identity to each other, with the full conservation of 6 cysteine residues for disulfide bond formation. Interestingly, among all CART peptides, CART1, CART3, and CART5 peptides seem to be much more conserved among species than the other three CART peptides (CART2, CART4, CART6). Furthermore, some signature motifs unique to certain types of CART peptides, such as ‘RCY’ in CART5 peptide and ‘KCI’ in CART4 peptide, were also identified (Fig 2, S1 and S2 Figs).

### Tissue expression of chicken *CART1* gene

**Fig 4:**
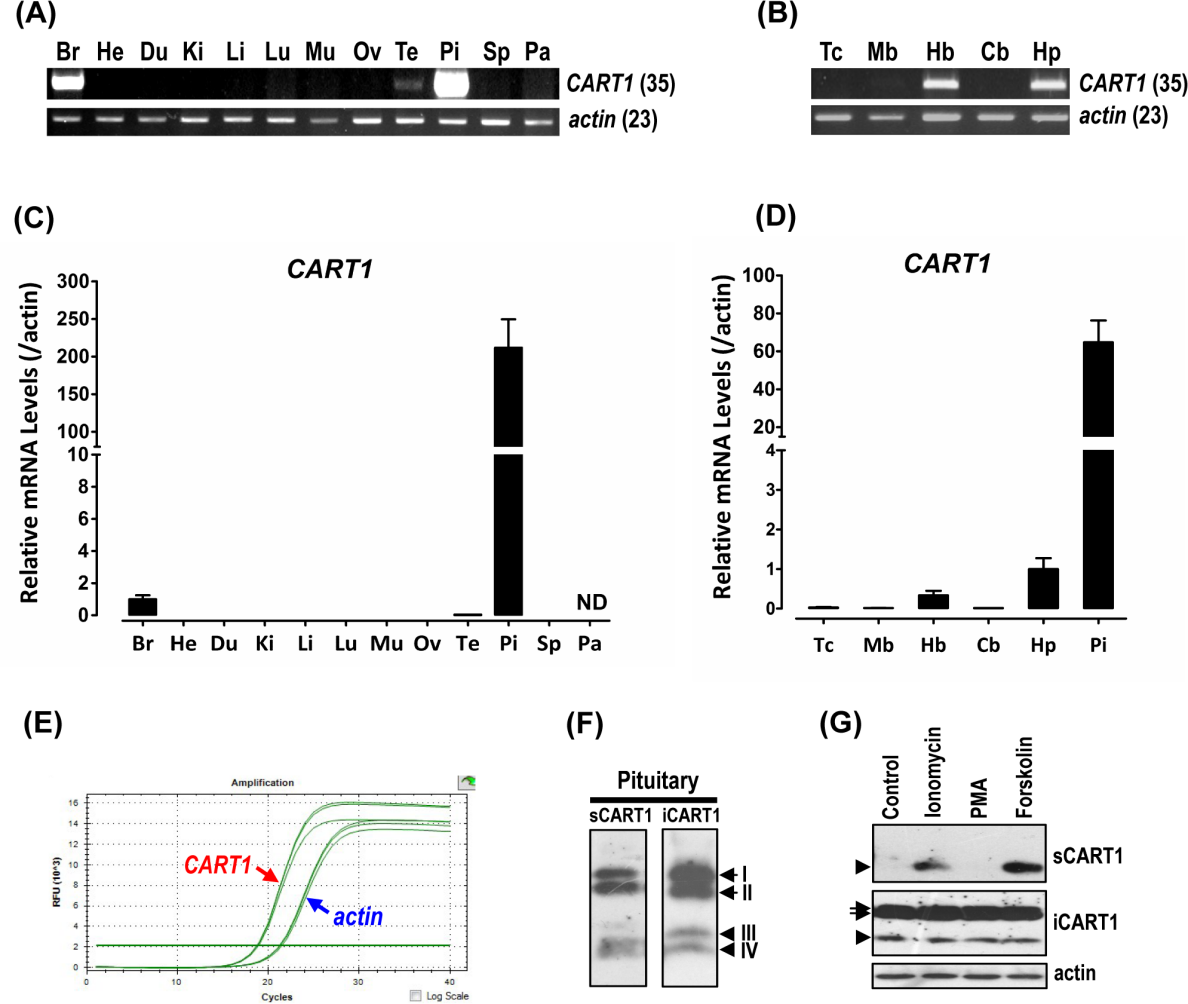
Expression of *CART1* in chicken tissues. RT-PCR detection of *cCART1* mRNA expression in (**A**) adult chicken tissues including the whole brain (Br), heart (He), duodenum (Du), kidneys (Ki), liver (Li), lung (Lu), muscle (Mu), ovary (Ov), testes (Te), pituitary (Pi), spleen (Sp) and pancreas (Pa); (**B**) various adult chicken brain regions including the telencephalon (Tc), midbrain (Mb), cerebellum (Cb), hindbrain (Hb) and hypothalamus (Hp). Numbers in brackets indicate the numbers of PCR cycles used. Quantitative RT-PCR assay of *cCART1* mRNA expression in (**C**) adult chicken tissues; (**D**) various adult brain regions and the pituitary. In (**C**) and (**D**), the mRNA levels of *CART1* genes were normalized to that of *β-actin* and expressed as the fold difference compared with that of the whole brain (Br) or hypothalamus (Hp). Each data point represents the mean ± SEM of 6 individual adult chickens (*N* = 6). ND indicates that *CART1* mRNA is undetectable in this tissue. (**E**) Amplification plot of *cCART1* and *β-actin* genes using the same amount of adult pituitary cDNA template. (**F**) Western blot detection of the 4 major CART1-ir bands (named CART1-I, II, III, IV) in adult chicken anterior pituitary tissue lysate (iCART1 means intracellular CART1), or in the medium (sCART1 means secreted CART1) of adult pituitaries incubated *in vitro* for 4 h. (**G**) Western blot detection of the effect of pharmacological drugs (500 nM ionomycin, 100 nM PMA, and 5 μM forskolin) on the secretion of CART1 from 1-week-old chick anterior pituitaries incubated *in vitro*. Multiple CART1-ir bands, including the two large CART1 bands (cCART1-I and cCART1-II, indicated by arrows) and a small band (cCART1-III/cCART1-IV, denoted by arrow heads) were identified in pituitary tissue lysate (iCART1), whereas only the small band (CART1-III or CART1-IV) was identified in the incubation medium of pituitaries treated with ionomycin and forskolin.

To elucidate the roles of c*CART1* gene in chicken tissues, using RT-PCR, we examined the mRNA expression of c*CART1* in adult chicken tissues including different brain regions (telencephalon, midbrain, hindbrain, cerebellum, and hypothalamus). As shown in Fig 4A, strong PCR signal of c*CART1* was easily detected in the whole brain and pituitary, and only a weak PCR signal in the testes. Within the CNS, the strong PCR signal was easily detected in the hindbrain and hypothalamus, while in other brain regions, *cCART1* signal was hardly discernible (Fig 4B).

The restricted mRNA expression of *CART1* in chicken tissues was further confirmed by quantitative real-time PCR assay. Pituitary c*CART1* mRNA level is at least 200-fold higher than that in the whole brain, and 15,000-fold higher than that in the testes, while in other peripheral tissues, c*CART1* mRNA is almost undetectable (Fig 4C).

Within the CNS, *cCART1* mRNA is highly expressed in the hypothalamus and hindbrain, whereas in other brain regions, including telencephalon, midbrain, and cerebrum, only a low level of *cCART1* expression was detected. Intriguingly, it should be noted that *cCART1* mRNA level in adult pituitary is at least 60-fold and 190-fold higher than that in the hypothalamus and hindbrain, respectively (Fig 4D and 4E), further indicating the predominant expression of *cCART1* mRNA in the anterior pituitary.

### Expression and secretion of *CART1* peptide in chicken anterior pituitary

In this study, we also examined *cCART1* protein expression in the pituitary using Western blot. As shown in Fig 4F, four major bands of sizes ranging from ~4 to 10 kDa, named CART1-I, CART1-II, CART1-III, and CART1-IV, respectively were easily detected in adult pituitary tissue lysate (Fig 4F). In contrast, no visible band was detected in other chicken tissues examined including liver, duodenum, testes, and hypothalamus. This finding further supports the predominant expression of CART1 in chicken anterior pituitary.

The abundant expression of c*CART1* in pituitaries also points out a possibility that cCART1 may be a novel hormone secreted by anterior pituitary. To test this hypothesis, we examined whether cCART1 is secreted by intact pituitaries incubated *in vitro* for 4 h. As shown in Fig 4F, multiple bands of sizes varying from ~4 to 10 kDa were detected in the incubation medium of adult pituitaries.

Interestingly, no visible band was detected in the incubation medium of anterior pituitaries collected from 1-week-old chicks, though multiple bands were still detected in the pituitary tissue lysates; however, the small cCART1 band (CART1-III/CART1-IV) was easily detected in the incubation medium, when the chick anterior pituitaries were treated by ionomycin (500 nM, 4 h) and forskolin (5 μM, 4 h) (Fig 4G), which are capable of elevating intracellular [Ca^2+^] and cAMP levels, respectively. Collectively, these findings suggest that cCART1 is a peptide hormone secreted by the anterior pituitary, and its secretion is under the control of the secretagogue(s) capable of elevating the cAMP and calcium levels in pituitary cells.

### Tissue expression of chicken *CART2* gene

**Fig 5:**
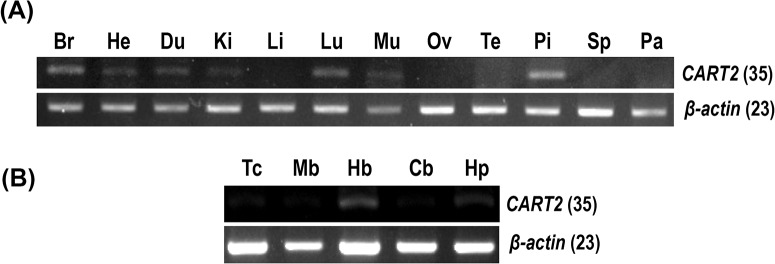
Expression of *CART2* gene in chicken tissues. **(A)** RT-PCR detection of *cCART2* mRNA expression in 12 adult chicken tissues including the whole brain (Br), heart (He), duodenum (Du), kidneys (Ki), liver (Li), lung (Lu), muscle (Mu), ovary (Ov), testes (Te), pituitary (Pi), spleen (Sp), and pancreas (Pa); (**B**) RT-PCR detection of mRNA expression of *cCART2* gene in various adult chicken brain regions including the telencephalon (Tc), midbrain (Mb), cerebellum (Cb), hindbrain (Hb), and hypothalamus (Hp). Numbers in brackets indicate the numbers of PCR cycles used.

Using RT-PCR, we also examined the *cCART2* mRNA expression in adult chicken tissues. Interestingly, unlike *cCART1*, only an extremely faint PCR signal of *cCART2* was detected in most tissues examined including heart, duodenum, kidney, lung, muscle, pituitary, telencephalon, midbrain, hindbrain, cerebellum, and hypothalamus, and it is undetectable in the liver, ovary, testes, spleen, and pancreas (Fig 5A and 5B). This finding implies a low level of *cCART2* expression in the chicken tissues examined.

### Expression profiles of *cCART1* gene in developing hypothalamus and pituitary

**Fig 6:**
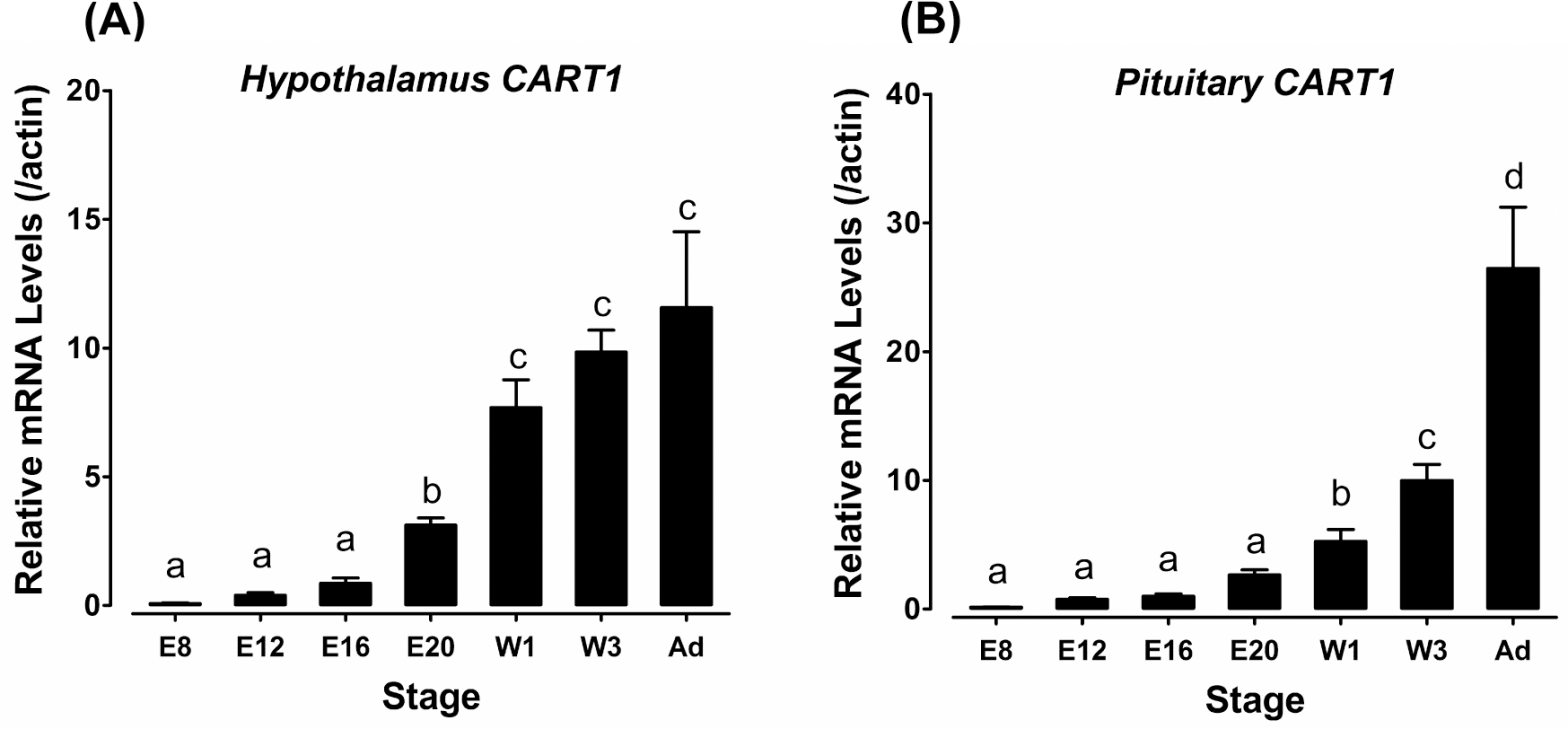
Expression of *CART1* in developing chicken hypothalami and pituitaries. Developmental expression of *cCART1* mRNA in the hypothalami (**A**) and pituitaries (**B**) of chicken embryos [embryonic day 8 (E8), day 12 (E12), day 16 (E16), and day 20 (E20)], 1-week (W1)-old and 3-week (W3)-old chicks, and adult chickens (Ad). In (**A**), and (**B**), the mRNA levels of all genes were examined by qRT-PCR. The relative mRNA level of *cCART1* gene was first calculated as the ratio to that of *β-actin* and then expressed as the fold difference compared to that of E16 chicken hypothalami or pituitaries. Each data point represent as means ± SEM of 6 individuals (*N* = 6). Values significantly different (*P* <0.05) between stages are indicated by different letters.

The relatively high mRNA levels of *cCART1* detected in adult chicken hypothalamus and pituitary also led us to further examine its expression in developing hypothalamus–pituitary axis from embryonic to adult stages using qRT-PCR. As shown in Fig 6, the *cCART1* mRNA level in the hypothalamus is low at embryonic day 8 (E8), E12, and E16, then increases significantly from the E20 to 1-week (w1) post-hatch stage, and is finally maintained at a high level from 3-week onto the adult stage. Similarly, the *cCART1* mRNA level in the pituitary is low at E8, E12 and E16, and then increases at E20. After hatching, pituitary *cCART1* mRNA level further increases at 1-week and 3-week post-hatch stages and reaches its maximum at the adult stage.

### Effect of fasting on *cCART1* mRNA expression in chicken hypothalamus and pituitary

**Fig 7:**
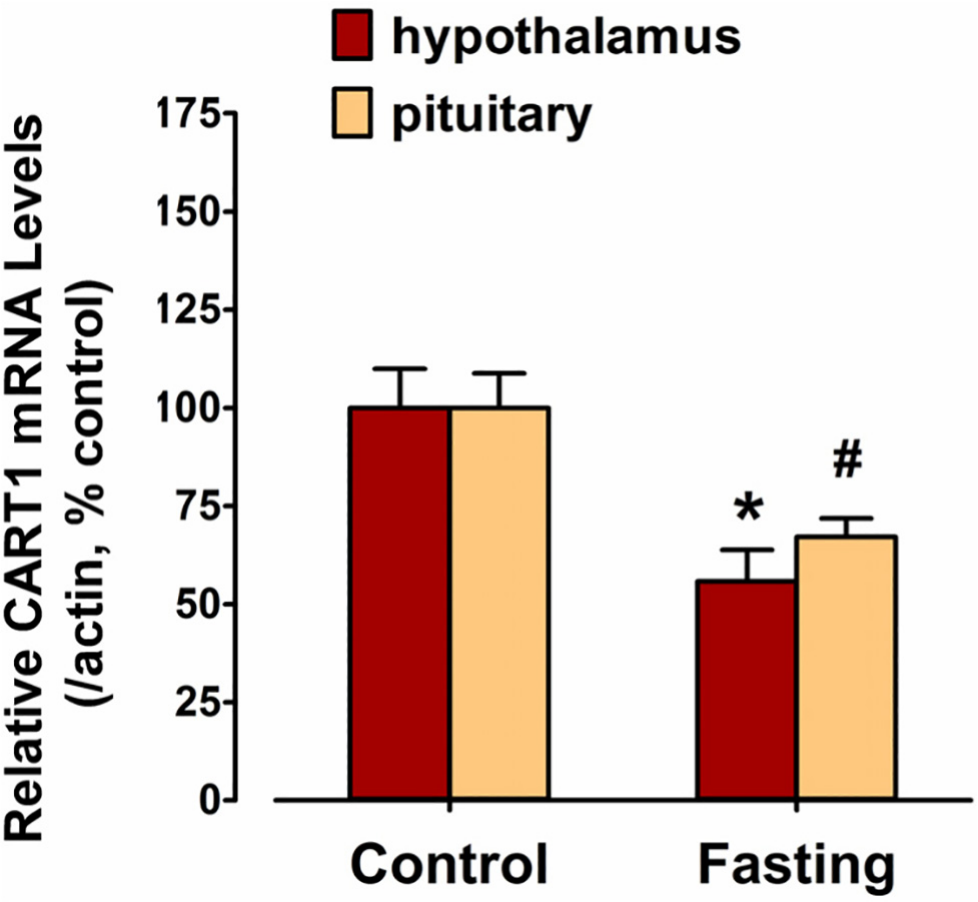
Fasting downregulates *cCART1* expression in chick hypothalamus and pituitary. The effect of 48-h fasting on the *cCART1* mRNA expression in the hypothalami and pituitaries of 2-week-old chicks examined by qRT-PCR. The relative mRNA level of *cCART1* gene was first calculated as the ratio to that of *β-actin* and then expressed as the percentage compared to that of control group chicks with free access to food and water. Each data point represent as means ± SEM of 10 individuals (*N* = 10). *, #, *P* < 0.01 *vs* control.

As shown in Fig 7, fasting for 48 hours reduces *cCART1* mRNA level in the hypothalamus of 2-week-old male chicks significantly to 55.8% of that detected in the control. Similarly, the *cCART1* mRNA level also shows a significant decrease in the pituitary after 48-h fasting (67.1% of the control).

## Discussion

In this study, two *CART* genes, named c*CART1* and c*CART2* respectively, are identified and characterized in chickens. *cCART1* is predominantly expressed in the anterior pituitary, and less abundantly in the hypothalamus, and its expression at both sites is down-regulated by fasting, while *cCART2* mRNA is only weakly expressed in chicken tissues examined. These findings not only imply an association of *cCART1* expression with food deprivation, but also represent the first report that c*CART1* is a novel peptide hormone secreted by anterior pituitary in chickens. As in chickens, 2 to 7 *CART* genes are identified in non-mammalian vertebrates via data mining of genome database. The identification of the repertoire of *CART* genes in representative vertebrate species including chickens would undoubtedly establish a clear basis to interpret the physiological roles of *CART(s)* played in different vertebrates and their evolutionary relationship.

### Identification of two *CART* genes in chickens

CART peptide is implicated in inhibition of food intake in chickens [39], however, the information regarding the structure and expression of chicken *CART* gene(s) is unclear. In this study, two *CART* genes (*CART1* and *CART2*) were cloned from chicken brain. Among them, only c*CART1* is orthologous to the mammalian *CART* gene, and the predicted bioactive cCART1(49–89) shares remarkable degree of identity (98%) with human or rat CART(49–89). Since the dibasic residues (K^40^R^41^, K^47^K^48^) critical for proteolytic processing are fully conserved between chicken and mammalian proCARTs [48,49], therefore, it is most likely that as in mammals, the two bioactive CART1 peptides [cCART1(42–89) and cCART1(49–89)] are also produced in chickens. This idea was substantiated by the detection of two bands of expected sizes (cCART1-III, ~ 5.2 kDa; cCART1-IV, ~4.4 kDa) in adult chicken anterior pituitary lysate (Fig 4).

Interestingly, in addition to *cCART1*, a novel *CART2* gene was also identified in chickens in this study. Since the putative bioactive cCART2(51–91) shows only 59% identity with chicken CART1(49–89) or human CART(49–89), therefore, it remains unclear whether cCART2(51–91) plays a role either similar to, or distinct from, that of cCART1(49–89) played in chickens.

### Identification of multiple *CART* genes in non-mammalian vertebrates: nomenclature for *CART* genes in vertebrates

The identification of *CART1* and *CART2* in chickens also led us to examine the existence of the two *CART* genes in the genomes of other vertebrates, including coelacanth. Strikingly, in addition to *CART1* and *CART2* genes, four other *CART* genes, named *CART3*, *CART4*, *CART5* and *CART6*, respectively, were also identified in coelacanth genome. Among them, *CART6* gene has not been reported in any vertebrate species. Like the mammalian *CART* gene, all 6 coelacanth *CART* genes consist of three exons and two introns within the coding region [30]. Moreover, all proCARTs were predicted to be capable of generating the bioactive CART peptides of 41 or 48 amino acids after cleavage from the conserved dibasic residues (KR and/or KK). Interestingly, 2 to 7 *CART* genes orthologous to coelacanth *CART1-6* genes are also identified in the genomes of some teleosts or tetrapods (but not mammalian species) (Fig 3, Table 1).

**Fig 8:**
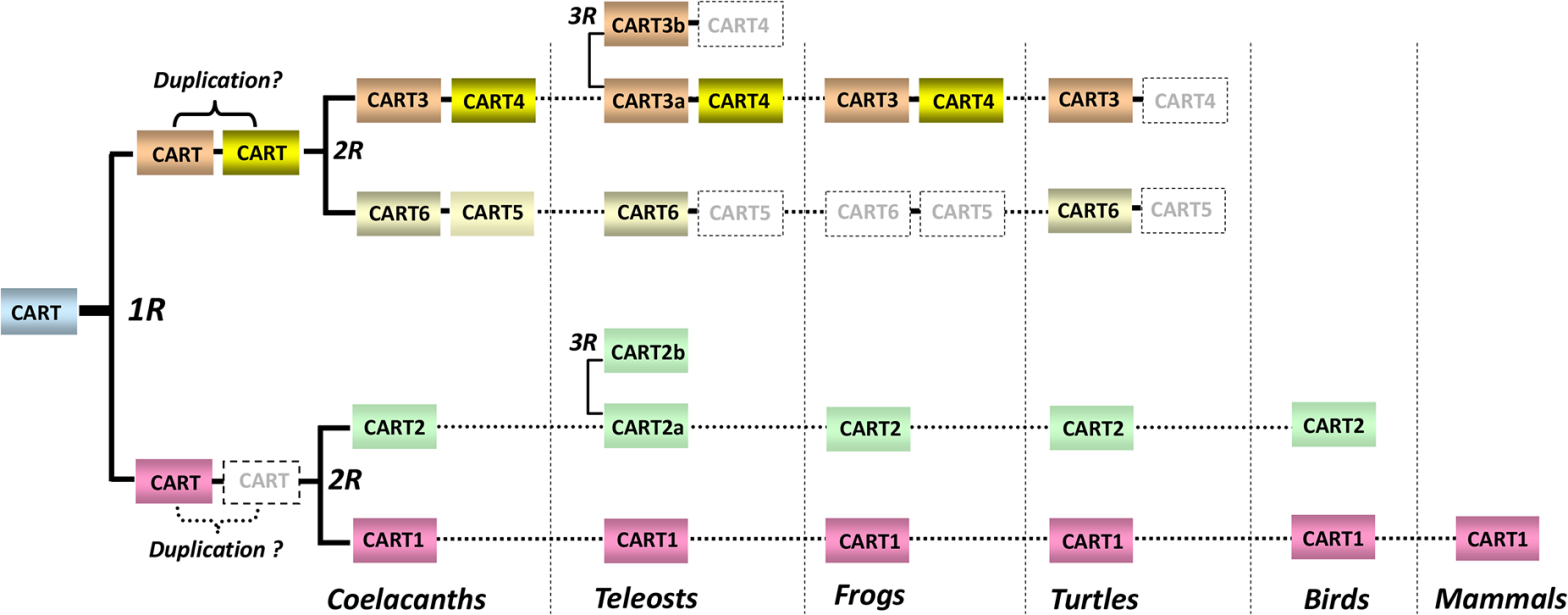
The proposed evolutionary history of vertebrate *CART* genes. A single ancestral *CART* gene may have given rise to a repertoire of multiple *CARTs* (≥ 6 *CARTs*) in the common ancestor of tetrapods and teleost fish after a local gene duplication and two whole genome duplication events (2R hypothesis) [50,51]. The third round of genome duplication event (3R) [47], or the lineage-specific gene losses may have resulted in the varying numbers of *CART* genes in coelacanths (6 *CARTs*), teleosts (*e*.*g*. 7 *CARTs* in tilapia), frogs (*e*.*g*. 4 *CART*s in *Xenopus tropicalis*), turtles (*e*.*g*. 4 *CARTs* in painted turtle), birds (*e*.*g*. 2 *CARTs* in chickens), and mammals (*e*.*g*. 1 *CART* in humans). Dotted boxes represent putative lost genes, or genes yet to be found. The question remains whether the local gene duplication event (tandem duplication) occurred before or after the 1st round genome duplication (**1R**) during vertebrate evolution and thus it is indicated by a question mark. The proposed scenario was based on the synteny analysis (Fig 3), amino acid sequence identity between CART peptides (Figs 1 and 2, S1 and S2 Figs), and the phylogenetic tree constructed by Maximum-likelihood method (S3 Fig). Future identification of *CART* genes from lower vertebrate species would broaden the dataset and further substantiate this hypothesis. 1R and 2R represent the first and second rounds of genome duplication events occurred in the early history of vertebrates respectively.

The identification of multiple homologous *CART* genes in non-mammalian vertebrates, together with their non-identical chromosomal localization shown in Fig 3, led us to hypothesize that all *CART* genes were derived from a common ancestral *CART* gene, which likely experienced both local gene duplication and 2 rounds of genome duplication events (2R hypothesis) occurred in the early history of vertebrate evolution (Fig 8) [50,51]. This hypothesis was supported by the identification of several paralogous genes [*e*.*g*. microtube-associated proteins (*MAP1A*, *MAP1B* and *MAP1S*); CREB regulated transcription coactivators (*CRTC2* and *CRTC3*)] adjacent to these *CART* genes in coelacanth and human genomes (Fig 3), as well as the evolutionary relationship of these *CART* genes as revealed by our phylogenetic analysis (S3 Fig). Moreover, the third round of genome duplication (3R) event, which occurred in the stem lineage of ray-finned fishes around ~350 mya ago [47], resulted in the emergence of two copies of *CART2* (*CART2a* and *CART2b*) and *CART3* (*CART3a* and *CART3b*) in teleost fishes including tilapia and zebrafish (Fig 3). Our hypothesis is different from the evolutionary scenario of *CART* genes in vertebrates proposed by Murashita *et al* (2009), which suggest the role of genome duplication, but not the local gene duplication, in the generation of multiple *CART* genes during vertebrate evolution [36]. Although 6 *CART* genes are proposed to exist in the last common ancestor of tetrapods and teleosts (Fig 8), lineage-specific gene losses during evolution may result in only 4 *CART* genes identified in turtles and frogs, 2 *CART* genes in birds, and a single *CART* gene in mammals.

The existence of multiple *CART* genes in teleosts (6–7 *CART*s), coelacanths (6 *CART*s), frogs (4 *CART*s), turtles (4 *CART*s), and birds (2 *CART*s) also substantiate the need to review the current nomenclature for *CART* genes in vertebrates. For instance, in medaka, 6 *CART* genes were identified and named as CART ch3, CART ch4, CART ch6, CART ch9, CART ch11, and CART-ch22 respectively based on their chromosomal location [38]. In zebrafish, only four *CART* genes have been found and they were named as *CART1*, *CART2a*, *CART2b*, and *CART3* respectively in one study [37], or *CART1-4* in another [52]. The inconsistent nomenclature will undoubtedly prevent our better understanding of the evolutionary relationship between these CART genes and the roles of each *CART* gene played in vertebrates. In this study, we identified 6 *CART* genes in the genome of coelacanth, a primitive lobe-finned fish. Since 6 *CART* genes orthologous to coelacanth *CART1-6* could still be identified in teleost fish or tetrapod species by synteny analysis (Fig 3), therefore, we suggest that *CART* gene(s) identified in vertebrates, particularly in non-mammalian vertebrates, are better to be named as *CART1*, *CART2*, *CART3*, *CART4*, *CART5*, *or CART6*, according to their orthology to the corresponding coelacanth *CART* genes (Fig 3 **)**. Based on this idea, we recommended the following nomenclature for these vertebrate *CART* genes: 1) the single *CART* gene in mammals is re-named as *CART1*, because it is the first *CART* gene identified in vertebrates, which is also orthologous to *CART1* gene identified in chickens and coelacanths; 2) 7 *CART* genes identified in Nile tilapia are named as *CART1*, *CART2a*, *CART2b*, *CART3a*, *CART3b*, *CART4*, and *CART5*, respectively according to their orthology to the corresponding coelacanth *CART* genes shown in Fig 3. Similarly, the 4 *CART* genes reported in zebrafish [37], originally called *CART1*, *CART2a*, *CART2b*, and *CART3*, are suggested to be re-named as *CART2b*, *CART3b*, *CART3a*, and *CART5* respectively, because *CART1* gene orthologous to coelacanth and human *CART1* is absent in zebrafish genome, as indicated by synteny analysis (Fig 3A). Furthermore, two novel *CART* genes, named *CART2a* and *CART4* respectively, were identified in zebrafish genome in the present study, although their expression has not yet been investigated. The suggested nomenclature for all *CART* genes identified in other representative vertebrate species, including frogs and turtles, is listed in Table 1. Clearly, this nomenclature will undoubtedly help us to interpret the physiological roles of each CART played in different classes of vertebrates [38].

In mammals, the biological actions of CART peptide are proposed to be mediated by the unidentified CART receptor [30,53], or perhaps by multiple CART receptors. The existence of 2–7 *CART* peptides encoded by separate genes in non-mammalian vertebrates also tend to support the existence of multiple CART receptor genes in non-mammalian vertebrates, which may be capable of mediating the similar, but non-identical, physiological roles of distinct CART peptides in non-mammalian vertebrates. Clearly, the identification of the receptor(s) for 2 to 7 CART peptides encoded by different genes in non-mammalian vertebrates will substantiate our hypothesis.

### Tissue expression of *cCART1* gene: Evidence for CART1 being a novel hormone secreted by anterior pituitary

In this study, within the CNS of chickens, *cCART1* mRNA has been found to be highly expressed in the hypothalamus, moderately in hindbrain, and only weakly in other brain regions. This finding is consistent with the findings in rats and several teleost fishes, in which *CART* is also expressed in the hypothalamus and hindbrain [9,16,37,52,54]. The expression of c*CART1* in chicken hypothalamus also supports roles of cCART1 played in the hypothalamus, such as inhibition of food intake as previously reported [39].

Strikingly, in spite of the high *cCART1* mRNA levels detected in chicken hypothalamus, *cCART1* mRNA level in adult chicken pituitary is found to be 60-fold higher than that in hypothalamus. This finding, together with the little or no expression of *cCART1* mRNA in peripheral tissues, clearly indicates that unlike that in mammals and teleosts [38], the pituitary, but not hypothalamus (or brain), is the major source of cCART peptide in chickens. Furthermore, we noted that *cCART1* mRNA levels in the pituitary seem to be even much higher than that of *β-actin*, as roughly estimated by their amplification plots shown in Fig 4E, in which Ct value of *cCART1* is even lower than that of *β-actin* under the experimental condition. The incredibly high mRNA levels of *CART1* noted in chicken pituitary led us to hypothesize that CART1 is most likely to be a novel peptide hormone secreted by the anterior pituitary. In agreement with this idea, CART1 peptide was easily detected in pituitary and its secretion from 1-week chick pituitaries incubated *in vitro* was induced by forskolin and ionomycin. This finding clearly indicates that cCART1 secretion is controlled by secretagogue(s), which can elevate the cAMP and/or calcium signaling pathway(s) in pituitary cells. As in chickens, *CART* mRNA and protein is reported to be expressed in the anterior pituitary in mammals [4,11,17,18]. In rats, CART-immunoreactivity is localized in several pituitary cell populations, including lactotrophs, somatotrophs, corticotrophs, and gonadotrophs [11,15,17], and its expression and secretion can be induced by hypothalamic factors, such as corticotrophin-releasing hormone (CRH) [17]. Moreover, plasma CART levels also display a diurnal variation, resembling that of plasma ACTH and corticosterone levels [17]. All these findings from rodents suggest that pituitary is a source of CART peptide and may contribute to the circulating CART levels [15,17,33]. As in chickens and mammals, the mRNA expression of *CARTs* could also be detected in goldfish pituitary [35]. Together, these findings suggest that in addition to being a neuropeptide functioning in the CNS and PNS, pituitary CART may act as a novel endocrine (autocrine/paracrine) hormone to participate in many physiological processes of vertebrates as well. The predominant and abundant expression of *cCART1* in chicken pituitaries provides us a unique platform to reveal whether CART1 is a novel circulating hormone secreted from anterior pituitary and to uncover its endocrine actions on peripheral tissues, which still await both extensive and intensive studies in vertebrates [29].

In this study, 4 major CART-immunoreactive bands, named cCART1-I, cCART1-II, cCART1-III, and cCART1-IV, were detected in adult chicken pituitary tissue lysate. This finding is consistent with the findings in mammals, in which 5 CART bands in the range of 4–10 kDa were detected in rat hypothalamus and pituitary by Western blot [49]. The two small bands (cCART1-III and cCART1-IV) may correspond to the bioactive cCART1 peptides [cCART1(42–89), cCART1(49–89)] cleaved from the conserved dibasic residues (K^40^R^41^, K^47^K^48^), as reported in mammals [49], while the other two large bands (CART1-III and CART1-IV) may represent the proCART1, or the intermediate form(s) of proCART1 cleaved from the other dibasic/monobasic residue(s) (*e*.*g*. R^7^R^8^, R^10^, R^22^) (Fig 1) [4,49,55]. Intriguingly, multiple bands, including the two large CART bands (CART1-I and CART1-II) and small CART bands, were easily detected in the incubation medium of adult chicken anterior pituitary cultured *in vitro*. This finding further supports our hypothesis that cCART1 is a secretory pituitary hormone. However, unlike that in adult pituitary, only three major CART bands (cCART1-I, cCART1-II, and cCART1-III/cCART1-IV) were detected in one-week chick pituitaries incubated *in vitro*. Moreover, chick pituitaries seem to preferentially secrete the small bioactive CART band(s) [CART1(42–89) or CART1(49–89)] in response to drug treatment. The age-dependent secretion of CART peptides of different lengths from chicken pituitaries is interesting, and probably involved the proteolytic processing of the two pro-hormone convertases, PC2 and PC1/3 [48,56]. Both *in vivo* and *in vitro* studies have proven that PC2 is efficient in generating the small bioactive CART peptides in rats, while PC1/3 is predominantly active in liberating the intermediate CART forms [48]. The differential expression of PC2 and PC1/3 genes at different stages of chicken pituitaries demonstrated in our preliminary study seems to support their involvement in age-dependent pro-cCART1 processing.

In this study, we noted that c*CART1* expression in the pituitary and hypothalamus is stage-dependent. The *cCART1* mRNA levels at both sites are much lower during early embryogenesis, but increase steadily towards the end of incubation, indicating it probably has a yet identified role in late embryogenesis. After hatching, the *cCART1* mRNA expression increases significantly in the hypothalamus and pituitary, and is maintained at high levels from one-week to the adult stage. The high *cCART1* mRNA levels noted at both sites also support a more important role of *cCART1* played in chicken hypothalamus-pituitary axis at post-hatch stage.

Unlike *cCART1*, *cCART2* mRNA was detected to be weakly expressed in most tissues examined, including the hypothalamus and pituitary. This finding, together with the limited identity (59%) shared between cCART1 and cCART2 peptides, also cast doubt on whether cCART2 plays a substantial role in chickens. Clearly, more studies are required to address this issue.

### The association of *cCART1* expression in the hypothalamus and pituitary with fasting

In this study, we noted that 48-h fasting downregulates the *cCART1* mRNA expression in the hypothalamus of 2-week-old layer chicks. This observation contrasts to the finding in a previous study, in which *cCART1* mRNA levels in the hypothalamus show no variation in 1-week-old broiler chicks after 48-h fasting [57]. A possible explanation for this discrepancy is due to different chicken strains used in two studies. However, our finding is consistent with the findings in rodents [5,58], goldfish [59], Atlantic cods [60], channel catfish [61], Atlantic salmon [36], medaka [62], and zebrafish [37], in which food-deprived animals show a significant reduction in their *CART(s)* mRNA levels in the hypothalamus (or brain). All these findings support an association of *CART* expression in the hypothalamus (or brain) with nutritional status in vertebrates including chickens.

In mammals, leptin, an adipocyte-derived hormone that signals energy store levels to the hypothalamus, has been proven to be a critical factor involved in the regulation of CART expression in the hypothalamus, particularly in the arcuate nucleus [5,8,58]. Peripheral administration of leptin to obese mice induces *CART* mRNA expression, while *CART* mRNA is almost absent in the arcuate nucleus of leptin-deficient (*ob*) mice or leptin receptor (*fa*)-deficient rats [5]. Recently, the authentic leptin (*ob*) gene has been identified in several birds, including zebra finches and pigeons by our and other laboratories [63–67]. Interestingly, we noted that avian leptin mRNA is predominantly expressed in the brain and pituitary, but undetectable in peripheral tissues including adipose tissue [63]. This finding, together with little effect of icv injection of leptin on food intake of chickens [68,69], also points to a possibility that unlike that in mammals, metabolic signal(s) other than leptin associated with negative energy balance from peripheral tissues may control c*CART1* mRNA expression in chicken hypothalamus (Fig 6).

Interestingly, fasting also reduces the c*CART1* mRNA expression in the anterior pituitary. This finding has not been reported in any vertebrate species. Considering the diverse actions of CART in the CNS, PNS, as well as in peripheral tissues, such as modulation of pancreatic functions and ovarian development and functions in mammals [23,24], it led us to hypothesize that the down-regulation of pituitary *CART1* expression may decrease the plasma cCART1 levels and thus attenuate the actions of CART1 on target tissues when calorie intake is restricted. Future studies on plasma cCART1 levels at different nutritional status and its actions on chicken tissues will provide critical clues to this matter.

In summary, two *CART* genes, c*CART1* and c*CART2*, were identified in chickens. *CART1* is shown to be predominantly expressed in the pituitary, and less abundantly in the hypothalamus and hindbrain. Evidence presented here strongly suggests that cCART1 is a novel hormone secreted by anterior pituitary. In addition, fasting was demonstrated to reduce *cCART1* mRNA expression in both the pituitary and hypothalamus, implying an association of *cCART1* expression with negative energy balance. Unlike *cCART1*, *cCART2* is only weakly expressed in chicken tissues. As in chickens, 2–7 *CART* genes, likely generated by both local gene duplication and genome duplication, are identified in other non-mammalian vertebrates in this study. The identification of the multiple *CART* genes in representative vertebrate species including chickens, would undoubtedly establish a clear basis to interpret the physiological roles of these structurally related peptides played in different classes of vertebrates.

## Supporting Information

S1 Fig Amino acid sequence alignment of vertebrate CART3/CART4. **(A**) Amino acid sequence alignment of painted turtle CART3 precursor (XM_005300628.2) with that of *Xenopus tropicalis* (NM_001079103), zebrafish (CART3b: NM_001017570; CART3a: GU057835), Nile tilapia (CART3a: XM_003456893; CART3b: XM_003449187), takifugu (CART3a: XM_003969743; CART3b: XM_003967297), tetraodon, medaka (CART3a: AB568292; CART3b: AB568294) and coelacanths (XM_005989732), or with that of CART1 precursor of chickens (KC249966) and humans (NM_004291). (**B**) Amino acid sequence alignment of *Xenopus tropicalis* CART4 precursor (accession no.: XM_002932246) with that of tilapia (XM_005461629), tetraodon (CAAE01014596), takifugu, zebrafish, elephant shark (XM_007908392), bicolor damselfish (XM_008296027), spotted gar (XM_006628839), and coelacanths. The conserved dibasic residue (KR/KK) for proteolytic processing is indicated by two arrows presented in proCARTs. Lines linking cysteines indicate the three disulfide bonds. Asterisks indicating the signature motif possessed by vertebrate CART4. All CART sequences from other species were either predicted according to their genomic sequence, or retrieved from GenBank.
(PDF)

S2 Fig Amino acid sequence alignment of vertebrate CART5/CART6. **(A**) Amino acid sequence alignment of zebrafish CART5 precursor (NM_001082932) with that of takifugu (XM_003969141), medaka (AB568296), Nile tilapia (XM_003455220), tetraodon, Mexican tetra (XM_007245650), platyfish (XM_005813911), amazon molly (XM_007566522), bicolor damselfish (XM_008304772), zebra mbuna (XM_004573662), and coelacanths. (**B**) Amino acid sequence alignment of green sea turtle CART6 precursor (EMP28211) with that of Burmese python (XM_007429116), and coelacanths. The conserved dibasic residue (KR and KK) for proteolytic processing is indicated by two arrows presented in proCARTs. Lines linking cysteines indicate the three disulfide bonds. Dots indicating the signature motif possessed by vertebrate CART5. All CART sequences from other species were either predicted according to their genomic sequence, or retrieved from GenBank.
(PDF)

S3 Fig Phylogenetic analysis. Phylogenetic tree (constructed by Maximum likelihood method) showing the evolutionary relationship of *CART* genes from non-mammalian and mammalian vertebrates. Numbers near each branch point indicates the bootstrap values. The amino acid sequence of all *CART* genes were either retrieved from GenBank or predicted according to genomic sequences.
(PDF)

S1 Table Primers used^a^. (PDF)
