# Supplementary material for: Characterization of the Two CART Genes (CART1 and CART2) in Chickens (Gallus gallus)
Source: PLoS One. 2015 May 18;10(5):e0127107. doi: 10.1371/journal.pone.0127107 (PMC4436185; doi:10.1371/journal.pone.0127107)
Supplement: S1 Fig — (A) Amino acid sequence alignment of painted turtle CART3 precursor (XM_005300628.2) with that of Xenopus tropicalis (NM_001079103), zebrafish (CART3b: NM_001017570; CART3a: GU057835), Nile tilapia (CART3a: XM_003456893; CART3b: XM_003449187), takifugu (CART3a: XM_003969743; CART3b: XM_003967297), tetraodon, medaka (CART3a: AB568292; CART3b: AB568294) and coelacanths (XM_005989732), or with that of CART1 precursor of chickens (KC249966) and humans (NM_004291). (B) Amino acid sequence alignment of Xenopus tropicalis CART4 precursor (accession no.: XM_002932246) with that of tilapia (XM_005461629), tetraodon (CAAE01014596), takifugu, zebrafish, elephant shark (XM_007908392), bicolor damselfish (XM_008296027), spotted gar (XM_006628839), and coelacanths. The conserved dibasic residue (KR/KK) for proteolytic processing is indicated by two arrows presented in proCARTs. Lines linking cysteines indicate the three disulfide bonds. Asterisks indicating the signature motif possessed by vertebrate CART4. All CART sequences from other species were either predicted according to their genomic sequence, or retrieved from GenBank. (PDF) [file pone.0127107.s001.pdf]

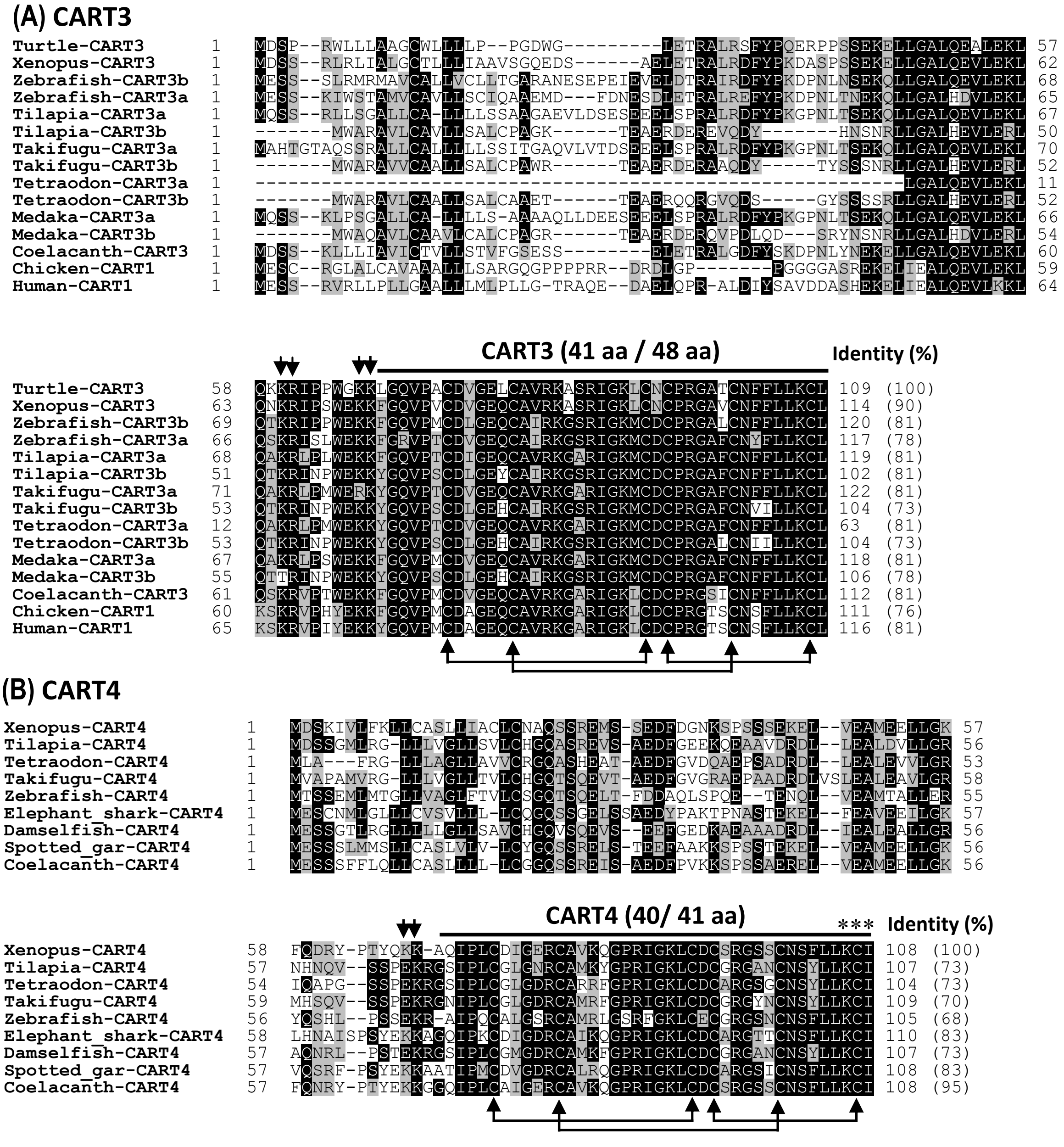
(A) CART3
Turtle-CART3 1 MDSP--RWLLLAAGCWLLLLP--PGDWG---------LETRALRSFYPQERPPSSEKELLGALQEALEKL 57
Xenopus-CART3 1 MDSS--RLRLIALGCTLLLIAAVSGQEDS-----AELETRALRDFYPKDASPSSEKELLGALQEVLEKL 62
Zebrafish-CART3b 1 MESS--SLRMRMAVCALLVCLLTGARANESEPEIEVELDTRAIRDFYPKDPNLNSEKQLLGALQEVLEKL 68
Zebrafish-CART3a 1 MESS--KIWSTAMVCAVLLSCIQAAEMD---FDNESDLETRALREFYPKDPNLTNEKQLLGALHDVLEKL 65
Tilapia-CART3a 1 MQSS--RLLSGALLCA-LLLLSSAAGAEVLDSESEEELSPRALRDFYPKGPNLTSEKQLLGALQEVLEKL 67
Tilapia-CART3b 1 -------MWARAVLCAVLLSALCPAGK------TEAERDEREVQDY-------HNSNRLLGALHEVLERL 50
Takifugu-CART3a 1 MAHTGTAQSSRALLCALLLLLSSITGAQVLVTDSEEELSPRALRDFYPKGPNLTSEKQLLGALQEVLEKL 70
Takifugu-CART3b 1 -------MWARAVVCAALLSALCPAWR------TEAERDERAAQDY-----TYSSSNRLLGALHEVLERL 52
Tetraodon-CART3a 1 ---LGALQEVLEKL 11
Tetraodon-CART3b 1 -------MWARAVLCAALLSALCAAET------TEAERQQRGVQDS-----GYSSSSRLLGALHEVLERL 52
Medaka-CART3a 1 MQSS--KLPSGALLCA-LLLLS-AAAAQLLDEESEEELSPRALRDFYPKGPNLTSEKQLLGALQEVLEKL 66
Medaka-CART3b 1 -------MWAQAVLCAAVLCALCPAGR------TEAERDERQVPDLQD---SRYNSNRLLGALHDVLERL 54
Coelacanth-CART3 1 MDSS--KLLLIAVICTVLLSTVFGSESS--------ELETRALGDFYSKDPNLYNEKELLGALQEVLEKL 60
Chicken-CART1 1 MESC--RGLALCAVAAALLLSARGQGPPPPRR--DRDLGP-------PGGGGASREKELIEALQEVLEKL 59
Human-CART1 1 MESS--RVRLLPLLGAALLLMLPLLG-TRAQE--DAELQPR-ALDIYSAVDDASHEKELIEALQEVLKKL 64
CART3 (41 aa / 48 aa)
Identity (%)
Turtle-CART3 58 QKKRIPPWGKKLGQVPACDVGELCAVRKASRIGKLCNCPRGATCNFFLLKCL 109 (100)
Xenopus-CART3 63 QNKRIPSWEKKFGQVPVCDVGEQCAVRKASRIGKLCNCPRGAVCNFFLLKCL 114 (90)
Zebrafish-CART3b 69 QTKRIPPWEKKFGQVPMCDLGEQCAIRKGSRIGKMCDCPRGALCNFFLLKCL 120 (81)
Zebrafish-CART3a 66 QSKRISLWEKKFGRVPTCDVGEQCAIRKGSRIGKMCDCPRGAFCNYFLLKCL 117 (78)
Tilapia-CART3a 68 QAKRLPLWEKKFGQVPTCDIGEQCAVRKGARIGKMCDCPRGAFCNFFLLKCL 119 (81)
Tilapia-CART3b 51 QTKRINPWEKKYGQVPSCDLGEYCAIRKGSRIGKMCDCPRGAFCNFFLLKCL 102 (81)
Takifugu-CART3a 71 QAKRLPMWERKYGQVPTCDVGEQCAVRKGARIGKMCDCPRGAFCNFFLLKCL 122 (81)
Takifugu-CART3b 53 QTKRINPWEKKYGQVPSCDLGEHCAIRKGSRIGKMCDCPRGAFCNVILLKCL 104 (73)
Tetraodon-CART3a 12 QAKRLPMWEKKYGQVPTCDVGEQCAVRKGARIGKMCDCPRGAFCNFFLLKCL 63 (81)
Tetraodon-CART3b 53 QTKRINPWEKKYGQVPSCDLGEHCAIRKGSRIGKMCDCPRGALCNIILLKCL 104 (73)
Medaka-CART3a 67 QAKRLPSWEKKYGQVPMCDVGEQCAVRKGARIGKMCDCPRGAFCNFFLLKCL 118 (81)
Medaka-CART3b 55 QTTRINPWEKKYGQVPSCDLGEHCAIRKGSRIGKMCDCPRGAFCNFFLLKCL 106 (78)
Coelacanth-CART3 61 QSKRVPTWEKKFGQVPMCDVGEQCAVRKGARIGKLCDCPRGSICNFFLLKCL 112 (81)
Chicken-CART1 60 KSKRVPHYEKKFGQVPMCDAGEQCAVRKGARIGKLCDCPRGTSCNSFLLKCL 111 (76)
Human-CART1 65 KSKRVPIYEKKYGQVPMCDAGEQCAVRKGARIGKLCDCPRGTSCNSFLLKCL 116 (81)
(B) CART4
Xenopus-CART4 1 MDSKIVLFKLLCASLLIACLCNAQSSREMS-SEDFDGNKSPSSSEKEL--VEAMEELLGK 57
Tilapia-CART4 1 MDSSGMLRG-LLLVGLLSVLCHGQASREVS-AEDFGEEKQEAAVDRDL--LEALDVLLGR 56
Tetraodon-CART4 1 MLA---FRG-LLLAGLLAVVCRGQASHEAT-AEDFGVDQAEPSADRDL--LEALEVVLGR 53
Takifugu-CART4 1 MVAPAMVRG-LLLVGLLTVLCHGQTSQEVT-AEDFGVGRAEPAADRDLVSLEALEAVLGR 58
Zebrafish-CART4 1 MTSSEMLMTGLLVAGLFTVLCSGQTSQELT-FDDAQLSPQE--TENQL--VEAMTALLER 55
Elephant_shark-CART4 1 MESCNMLGLLLCVSVLLL-LCQGQSSGELSSAEDYPAKTPNASTEKEL--FEAVEEILGK 57
Damselfish-CART4 1 MESSGTLRGLLLLLGLLSAVCHGQVSQEVS--EEFGEDKAEAAADRDL--IEALEALLGR 56
Spotted_gar-CART4 1 MESSSLMMSLLCASLVLV-LCYGQSSRELS-TEEFAAKKSPSSTEKEL--VEAMEELLGK 56
Coelacanth-CART4 1 MESSSFFLQLLCASLLLL-LCGGQSSREIS-AEDFPVKKSPSSAEREL--VEAMEELLGK 56
CART4 (40/ 41 aa)
***
Identity (%)
Xenopus-CART4 58 FQDRY-PTYQKK-AQIPLCDIGERCAVKQGPRIGKLCDCSRGSSCNSFLLKCI 108 (100)
Tilapia-CART4 57 NHNQV--SSPEKRGSIPLCGLGNRCAMKYGPRIGKLCDCGRGANCNSYLLKCI 107 (73)
Tetraodon-CART4 54 IQAPG--SSPEKRGSIPLCGLGDRCARRFGPRIGKLCDCARGSGCNSYLLKCI 104 (73)
Takifugu-CART4 59 MHSQV--SSPEKRGNIPLCGLGDRCAMRFGPRIGKLCDCGRGYNCNSYLLKCI 109 (70)
Zebrafish-CART4 56 YQSHL--PSSEKR-AIPQCALGSRCAMRLGSRFGKLCECGRGSNCNSFLLKCI 105 (68)
Elephant_shark-CART4 58 LHNAISPSYEKKAGQIPKCDIGDRCAIKQGPRIGKLCDCARGTTCNSFLLKCI 110 (83)
Damselfish-CART4 57 AQNRL--PSTEKRGSIPLCGMGDRCAMKFGPRIGKLCDCGRGANCNSYLLKCI 107 (73)
Spotted_gar-CART4 57 VQSRF-PSYEKKAATIPMCDVGDRCALRQGPRIGKLCDCARGSICNSFLLKCI 108 (83)
Coelacanth-CART4 57 FQNRY-PTYEKKGGQIPLCAIGERCAVKQGPRIGKLCDCSRGSSCNSFLLKCI 108 (95)

S1 Fig
