# Supplementary material for: Characterization of the Two CART Genes (CART1 and CART2) in Chickens (Gallus gallus)
Source: PLoS One. 2015 May 18;10(5):e0127107. doi: 10.1371/journal.pone.0127107 (PMC4436185; doi:10.1371/journal.pone.0127107)
Supplement: S2 Fig — (A) Amino acid sequence alignment of zebrafish CART5 precursor (NM_001082932) with that of takifugu (XM_003969141), medaka (AB568296), Nile tilapia (XM_003455220), tetraodon, Mexican tetra (XM_007245650), platyfish (XM_005813911), amazon molly (XM_007566522), bicolor damselfish (XM_008304772), zebra mbuna (XM_004573662), and coelacanths. (B) Amino acid sequence alignment of green sea turtle CART6 precursor (EMP28211) with that of Burmese python (XM_007429116), and coelacanths. The conserved dibasic residue (KR and KK) for proteolytic processing is indicated by two arrows presented in proCARTs. Lines linking cysteines indicate the three disulfide bonds. Dots indicating the signature motif possessed by vertebrate CART5. All CART sequences from other species were either predicted according to their genomic sequence, or retrieved from GenBank. (PDF) [file pone.0127107.s002.pdf]

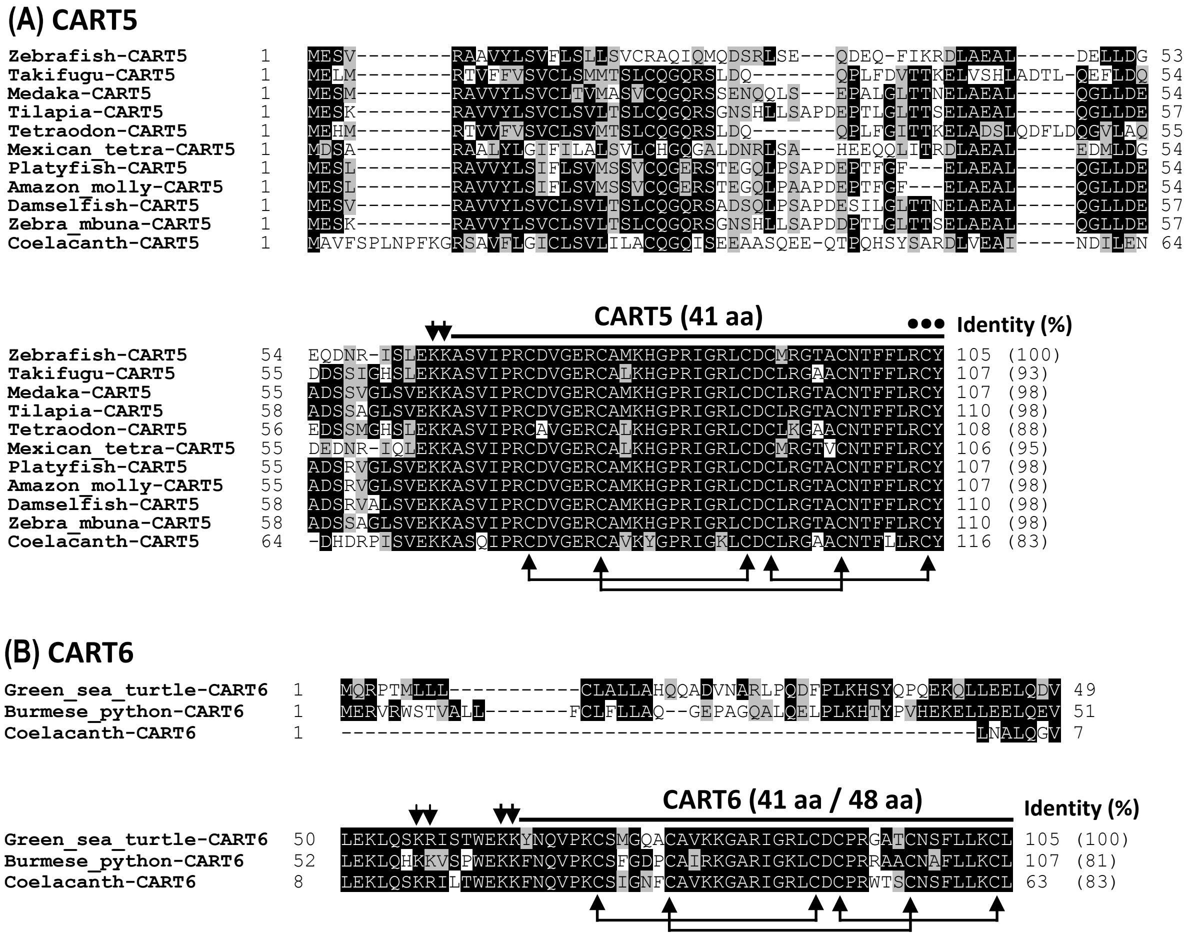
(A) CART5
Zebrafish-CART5 1 MESV--------RAAVYLSVFLSLLSVCRAQIQMQDSRLSE---QDEQ-FIKRDLAEAL-----DELLDG 53
Takifugu-CART5 1 MELM--------RTVFFVSVCLSMMTSLCQGQRSLDQ-------QPLFDVTTKELVSHLADTL-QEFLDQ 54
Medaka-CART5 1 MESM--------RAVVYLSVCLTVMASVCQGQRSSENQQLS---EPALGLTTNELAEAL-----QGLLDE 54
Tilapia-CART5 1 MESK--------RAVVYLSVCLSVLTSLCQGQRSGNSHLLSAPDEPTLGLTTSELAEAL-----QGLLDE 57
Tetraodon-CART5 1 MEHM--------RTVVFVSVCLSVMTSLCQGQRSLDQ-------QPLFGITTKELADSLQDFLDQGVLAQ 55
Mexican_tetra-CART5 1 MDSA--------RAALYLGIFILALSVLCHGQGALDNRLSA---HEEQQLITRDLAEAL-----EDMLDG 54
Platyfish-CART5 1 MESL--------RAVVYLSIFLSVMSSVCQGERSTEGQLPSAPDEPTFGF---ELAEAL-----QGLLDE 54
Amazon_molly-CART5 1 MESL--------RAVVYLSIFLSVMSSVCQGERSTEGQLPAAPDEPTFGF---ELAEAL-----QGLLDE 54
Damselfish-CART5 1 MESV--------RAVVYLSVCLSVLTSLCQGQRSADSQLPSAPDESILGLTTNELAEAL-----QGLLDE 57
Zebra_mbuna-CART5 1 MESK--------RAVVYLSVCLSVLTSLCQGQRSGNSHLLSAPDDPTLGLTTSELAEAL-----QGLLDE 57
Coelacanth-CART5 1 MAVFSPLNPFKGRSAVFLGICLSVLILACQGQISEEAASQEE-QTPQHSYSARDLVEAI-----NDILEN 64
CART5 (41 aa)
Identity (%)
Zebrafish-CART5 54 EQDNR-ISLEKKASVIPRCDVGERCAMKHGPRIGRLCDCMRGTACNTFFLRCY 105 (100)
Takifugu-CART5 55 DDSSIGHSLEKKASVIPRCDVGERCALKHGPRIGRLCDCLRGAACNTFFLRCY 107 (93)
Medaka-CART5 55 ADSSVGLSVEKKASVIPRCDVGERCAMKHGPRIGRLCDCLRGTACNTFFLRCY 107 (98)
Tilapia-CART5 58 ADSSAGLSVEKKASVIPRCDVGERCAMKHGPRIGRLCDCLRGTACNTFFLRCY 110 (98)
Tetraodon-CART5 56 EDSSMGHSLEKKASVIPRCAVGERCALKHGPRIGRLCDCLKGAACNTFFLRCY 108 (88)
Mexican_tetra-CART5 55 DEDNR-IQLEKKASVIPRCDVGERCALKHGPRIGRLCDCMRGTVCNTFFLRCY 106 (95)
Platyfish-CART5 55 ADSRVGLSVEKKASVIPRCDVGERCAMKHGPRIGRLCDCLRGTACNTFFLRCY 107 (98)
Amazon_molly-CART5 55 ADSRVGLSVEKKASVIPRCDVGERCAMKHGPRIGRLCDCLRGTACNTFFLRCY 107 (98)
Damselfish-CART5 58 ADSRVALSVEKKASVIPRCDVGERCAMKHGPRIGRLCDCLRGTACNTFFLRCY 110 (98)
Zebra_mbuna-CART5 58 ADSSAGLSVEKKASVIPRCDVGERCAMKHGPRIGRLCDCLRGTACNTFFLRCY 110 (98)
Coelacanth-CART5 64 -DHDRPISVEKKASQIPRCDVGERCAVKYGPRIGKLCDCLRGAACNTFLLRCY 116 (83)
(B) CART6
Green_sea_turtle-CART6 1 MQRPTMLLL-----------CLALLAHQQADVNARLPQDFPLKHSYQPQEKQLLEELQDV 49
Burmese_python-CART6 1 MERVRWSTVALL-------FCLFLLAQ--GEPAGQALQELPLKHTYPVHEKELLEELQEV 51
Coelacanth-CART6 1 -----------------------------------------------------LNALQGV 7
CART6 (41 aa / 48 aa)
Identity (%)
Green_sea_turtle-CART6 50 LEKLQSKRISTWEKKYNQVPKCSMGQACAVKKGARIGRLCDCPRGATCNSFLLKCL 105 (100)
Burmese_python-CART6 52 LEKLQHKKVSPWEKKFNQVPKCSFGDPCAIRKGARIGKLCDCPRRAACNAFLLKCL 107 (81)
Coelacanth-CART6 8 LEKLQSKRILTWEKKFNQVPKCSIGNFCAVKKGARIGRLCDCPRWTSCNSFLLKCL 63 (83)

**S2 Fig**
