# Supplementary figures and images for: Characterization of the Two CART Genes (CART1 and CART2) in Chickens (Gallus gallus)
Source: PLoS One. 2015 May 18;10(5):e0127107. doi: 10.1371/journal.pone.0127107 (PMC4436185; doi:10.1371/journal.pone.0127107)

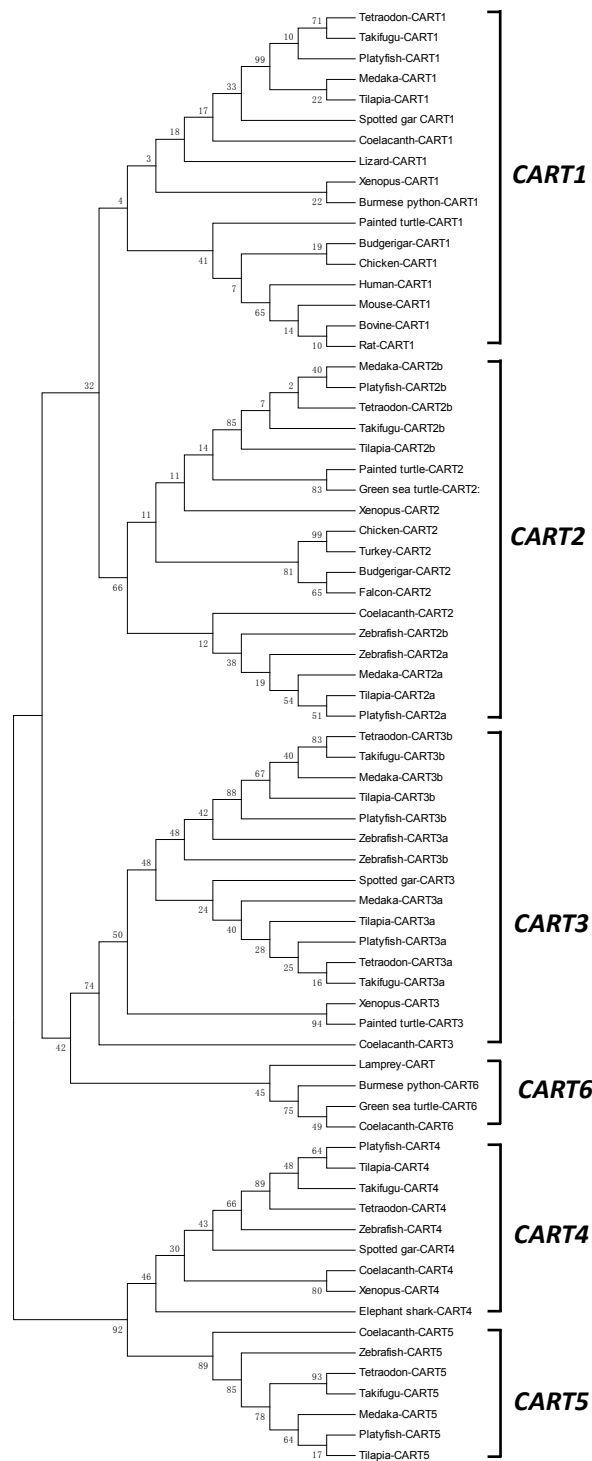

**S3 Fig**

Supplement: S3 Fig — Phylogenetic tree (constructed by Maximum likelihood method) showing the evolutionary relationship of CART genes from non-mammalian and mammalian vertebrates. Numbers near each branch point indicates the bootstrap values. The amino acid sequence of all CART genes were either retrieved from GenBank or predicted according to genomic sequences. (PDF) [file pone.0127107.s003.pdf]
