# Supplementary material for: Characterization of the Two CART Genes (CART1 and CART2) in Chickens (Gallus gallus)
Source: PLoS One. 2015 May 18;10(5):e0127107. doi: 10.1371/journal.pone.0127107 (PMC4436185; doi:10.1371/journal.pone.0127107)
Supplement: S1 Table — (PDF) [file pone.0127107.s004.pdf]

Table S1 *Primers used*[a]

Gene/construct	Sense/antisense	Primer sequence	Size (bp)
Primers for cloning the full-length cDNAs of CART1 and CART2			
cCART1	Sense	ACCATGGAGAGCTGCGGGGGCT	604
	Antisense	CGTCGGAGGGGCGAGCAGGAGGCGA	
cCART2	Sense	TGGGAAGATGGGCACTGCGTGGCT	387
	Antisense	ATCCCGGCAGACGGCAGCAGCCGT	
Primers for rapid amplification of 5'-cDNA ends of cCART2			
cCART2	Antisense	ATCCCGGCAGACGGCAGCAGCCGT	
	Antisense	AGACGGCAGCAGCCGTGGTGCCCCCA	
Primers for RT-PCR assay			
cCART1	Sense	ACCATGGAGAGCTGCGGGGGCT	604
	Antisense	CGTCGGAGGGGCGAGCAGGAGGCGA	
cCART2	Sense	GAAGATGGGCACTGCGTGGCTCT	300
	Antisense	CAGCTGCAGCGCTTCCCATAGCGA	
β-actin	Sense	TGTGCTACGTCGCACTGGAT	401
	Antisense	GCTGATCCACATCTGCTGGA	
Primers for quantitative real-time RT-PCR assay			
cCART1	Sense	CGTCCCGAGAGAAGGAGCTGATC	123
	Antisense	ACTGCTCTCCGGCGTCGCACAT	
β-actin	Sense	CCCAGACATCAGGGTGTGATG	123
	Antisense	GTTGGTGACAATACCGTGTTCAAT	

[a]All primers were synthesized by Beijing Genomics Institute (BGI, Shanghai, China).
